# Association between sense of coherence and loss of independence or death in older adults: Locomotive Syndrome and Health Outcomes in Aizu Cohort Study

**DOI:** 10.1371/journal.pone.0355726

**Published:** 2026-08-31

**Authors:** Sho Matsushita, Hajime Yamazaki, Yoshie Yamada, Yusuke Ogawa, Yosuke Yamamoto

**Affiliations:** 1 Department of Healthcare Epidemiology, Graduate School of Medicine, Kyoto University, Kyoto, Japan; 2 Department of General Internal Medicine, Hashimoto Municipal Hospital, Wakayama, Japan; 3 Section of Clinical Epidemiology, Department of Community Medicine, Graduate School of Medicine, Kyoto University, Kyoto, Japan; 4 Center for Innovative Research for Communities and Clinical Excellence (CiRC2LE), Fukushima Medical University, Fukushima, Japan; Aichi Medical University: Aichi Ika Daigaku, JAPAN

## Abstract

**Background:**

Although sense of coherence (SOC) is a core concept to promote healthy aging, evidence of whether SOC improves loss of independence (LOI) or death is lacking. We aimed to investigate the association between SOC and LOI or death in older adults.

**Methods:**

We enrolled independent older adults aged 65 years or over from a population-based cohort. SOC was assessed using a three-item SOC scale at baseline. The primary outcome was a composite of LOI (defined by Japanese long-term care insurance certification) or death. We investigated the association of SOC with LOI or death using the Cox proportional hazards model.

**Results:**

During a median follow-up of 5.8 years, 128 out of 1,661 (7.7%) participants had LOI or death. Lower SOC (per 1 standard deviation [SD] decrease) was associated with a higher risk of LOI or death (adjusted hazard ratio 1.25, 95% confidence interval [CI] 1.07 to 1.46, *p* = 0.005). Lower SOC (per 1 SD decrease) was associated with a higher risk of death (adjusted hazard ratio 1.24, 95% CI 1.03 to 1.50, *p* = 0.022) as well.

**Conclusions:**

Older adults with a lower SOC were more likely to experience LOI or death. Enhancing SOC could promote healthy aging in older adults.

## Introduction

Population aging is a global public health problem due to longer life expectancy and decreased fertility [1]. People aged 65 years or over accounted for 9.7% of the total global population in 2022—a percentage expected to increase to 16.4% in 2050 [2]. The aging population has led to an increased focus on the health of older adults [3]. The World Health Organization (WHO) defines health as ‘a state of complete physical, mental and social well-being’ [4]; however, this definition may not apply to older adults, many of whom face multiple chronic conditions [1]. As an alternative, it is proposed to redefine health as ‘the ability to adapt and manage oneself in the face of social, physical, and emotional challenges’ [5]. This revised definition places greater emphasis on subjective well-being, which involves emotional and cognitive evaluations of one’s own life.

The key factor for subjective well-being is sense of coherence (SOC), which refers to a pervasive, enduring, yet dynamic sense of confidence that life is comprehensible, manageable, and meaningful [6]. SOC consists of three components: comprehensibility, manageability, and meaningfulness [6]. Comprehensibility refers to the degree to which a person perceives stressful events as rational, predictable, structured, and understandable. Manageability refers to the belief that resources are available to address difficulties. Meaningfulness is defined as the belief that challenges in life are worthy of engagement [7,8]. Although SOC has traditionally been conceptualized as relatively stable after early adulthood, accumulating evidence suggests that it may be modifiable through life experiences and programs aimed at promoting self-care and self-management [9,10]. Older adults with high SOC can predict future events, manage events using internal and external resources, and attribute meaningful significance to them [8]. They can avoid stressful events and adapt to adversities such as chronic illness, physiological decline, and psychological stress [8,11]. Thus, people with a high SOC are considered to have lower mortality and less functional decline [12]. In fact, individuals with a high SOC have been found to be at a lower risk for poor medication adherence [13,14], incidence of diabetes [15], and development of cardiovascular diseases [16–18]. However, disability-free survival, defined as the composite of loss of independence (LOI) or death [19–22], has not yet been evaluated. Disability-free survival provides a more comprehensive assessment of overall health status because it captures not only mortality but also physical and mental disability, outcomes that are particularly relevant in older adults [19]. Therefore, the purpose of this study is to investigate, using a population-based cohort, whether SOC is associated with LOI or death in older adults.

## Methods

### Design and setting

This was a secondary analysis of data from the Locomotive Syndrome and Health Outcome in Aizu Cohort Study (LOHAS), a population-based prospective cohort study in Minami-Aizu Town and Tadami Town in Fukushima Prefecture, Japan. Tadami Town and Minami-Aizu Town are rural, mountainous regions, and the majority of participants were aged 70 years or older [23]. The LOHAS originally aimed to evaluate the association between locomotor dysfunction and the risk of cardiovascular disease. The study recruited community-dwelling participants aged 40–80 years who were National Health Insurance beneficiaries and participated in annual health checkups conducted by their local governments between 2008 and 2010 [23]. Survey forms were distributed to individuals attending the health checkups, and those who agreed to participate in the study returned the forms (the response rate was 60% in the first year) [23]. Approximately 5,800 individuals were recruited between 2008 and 2010 [24]. The baseline survey consisted of self-administered questionnaires, health checkups including blood and urine tests, musculoskeletal examinations, assessments of motor dysfunction, and radiographic assessments [23]. After the baseline examination, participants were followed prospectively, and the study is currently ongoing. The full LOHAS protocol has previously been published [23]. This study was approved by the ethics committees of Fukushima Medical University (673) and Kyoto University (R1730). Written informed consent was obtained from all the participants. Data were accessed for research purposes on 06/03/2023.

### Participants

The participants in the present study were community-dwelling older adults aged 65 years or over who underwent a baseline questionnaire survey in 2008. We excluded functionally dependent participants (any level of mandatory social long-term care insurance [LTCI] certification status) at baseline and participants with missing data on SOC and covariates. We described the baseline characteristics of participants with and without missing covariates. Statistical differences between these groups were assessed. Continuous variables were compared using the Wilcoxon rank-sum test, whereas categorical variables were compared using Pearson’s chi-square test. P values were presented for these comparisons.

### Sense of coherence (SOC)

SOC was assessed in 2008 using the University of Tokyo Health Sociology version of the SOC Scale (SOC-3-UTHS) [25]. The SOC-3-UTHS is a Japanese short version of the SOC scale, comprising three items that address manageability (“I am able to find solutions to the hardships and problems that occur every day”), meaningfulness (“I think it is worth facing and dealing with some of the hardships and problems of life”) and comprehensibility (“I am able to understand and predict the hardships and problems that occur every day”) [26]. Each item is rated on a 7-point scale, resulting in a total score range from 3 to 21. A higher score indicates a higher level of SOC [25]. The validity and reliability of SOC-3-UTHS have been shown in a previous study, reporting good internal consistency (Cronbach’s α = 0.84) and a moderate correlation with the SOC-13 (r = 0.51) [25]. A three-wave Japanese panel study supported the invariance of the factor structure of the SOC3-UTHS across time [27]. The stability coefficients between consecutive annual assessments were 0.69 and 0.70, suggesting that SOC was generally stable over time [27].

### Loss of independence (LOI) or death

We defined LOI as receiving care levels 3–5 in the LTCI certification status, in which people require complete support for many activities of daily living [28]. In care level 3, individuals cannot rise or walk; care level 4 means that they are nearly bedridden, and care level 5 signifies that they are completely bedridden [29]. We annually obtained information on LTCI certification status, including certified care levels and the dates of certification or subsequent updates, from administrative records maintained by the participating local governments. All Japanese people aged 65 years or over are eligible to apply for LTCI benefits based on physical and mental disabilities [30]. Older adults and their caregivers contact the municipal government, and the local government initially visit the home to assess nursing care needs. The Nursing Care Needs Certification Board, which includes physicians, nurses, and other health and social services experts, assigns LTCI certification status [30,31]. We did not obtain information about the exact cause of LOI; however, we evaluated each LOI case using the level of independence in daily living for patients with dementia in compliance with national government guidelines [32]. A score of level IIIa or higher on this scale indicates severe symptoms requiring care, which we categorized as severe dementia. We collected data on death dates and causes of death from death certificates. The incidence of LOI or death was evaluated from the day of baseline SOC measurement to the end of March 2014 or the day of relocation from Minami-Aizu Town or Tadami Town, which was censored.

### Covariates

As potential confounders, we adjusted for age, sex, body mass index, employment status (having a current full-time job, having a part-time job or other), living alone or not, number of comorbidities, alcohol consumption (none, less than 20 g/day, 20 g/day or more), smoking status (current smoker or not), baseline mental health status, and baseline physical activity. We assessed the number of comorbidities, including hypertension, diabetes, kidney disease, cerebrovascular disease, cardiovascular disease, respiratory disease, liver disease, and anemia (number of comorbidities: 0, 1, or ≥2). Mental health status was measured by Mental Health Inventory-5 (MHI-5) [33]. Physical activity was evaluated using the International Physical Activity Questionnaire as metabolic equivalents (min/week) [34].

### Statistical analysis

The median (interquartile range [IQR]) was used to describe baseline characteristics, and numbers and frequencies (percentages) were used to describe categorical variables. For the primary analysis, we investigated the association of SOC with LOI or death using Cox proportional hazards regression models to estimate hazard ratios (HRs) and 95% confidence intervals (CIs). Initially, we conducted a crude analysis, followed by an analysis adjusted for age and sex. We then performed multivariable analysis, adjusting for the abovementioned covariates. SOC was treated as a continuous variable in these analyses. We evaluated the Cox proportional hazards assumptions using Schoenfeld residuals.

We performed three sensitivity analyses. First, we additionally adjusted for marital status and the Social Functioning score of the 36-Item Short Form Health Survey (SF-36) to account for social factors in the relationship between SOC and the outcome [35]. These variables were not included in the primary analysis because social factors may act not only as confounders but also as potential mediators in the relationship between SOC and health outcomes, given the potentially bidirectional relationship between SOC and social factors [8]. Second, SOC was treated as a categorical variable. The SOC score was converted into a standardized score using the national mean SOC score from a previous national representative survey [36]. The standardized score was then categorized into three groups: the lowest SOC group (standardized score lower than 40), lower SOC group (standardized score ranging from 40 to 49), and high SOC group (standardized score ranging from 50 or higher, that is, equal to or exceeding the national mean score). We evaluated the association between SOC (a categorical variable) and LOI or death. Third, to address missing covariates data, we conducted multiple imputation analyses using the chained equation approach for 20 imputed datasets [37].

We further conducted two secondary analyses. First, we focused on death as the outcome. Cox proportional hazards models were used to estimate multivariable-adjusted HRs and 95% CIs for the association between SOC and death using the same covariates as the primary analysis. Second, we evaluated the association of SOC with the incidence of cause-specific mortality. Based on the International Statistical Classification of Disease and Related Health Problems, 10^th^ revision, we evaluated two major causes of death in Japan: cancer (all C-codes) and cardiovascular diseases (all I-codes).

We used STATA BE 17 (StataCorp, College Station, TX, USA) for all statistical analyses and reported 95% two-sided CIs [38].

## Results

### Participants’ characteristics

**Fig 1:**
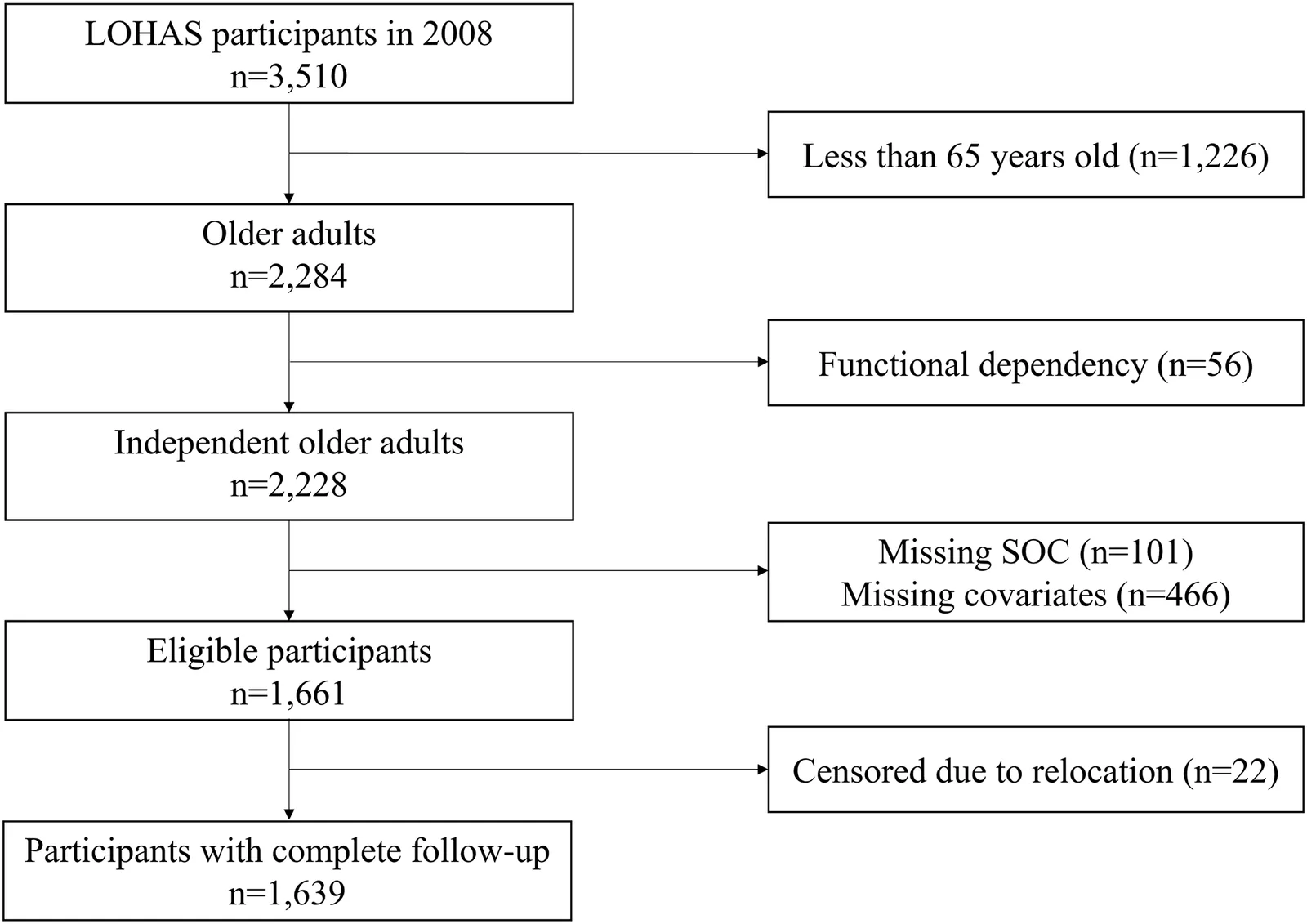
Study flow.

Among the 3,510 participants registered in the LOHAS, we identified 2,228 independent older adults in 2008. We excluded 101 participants with missing SOC measurements and 466 participants with missing covariate information. A total of 1,661 participants were eligible for this study (Fig 1). The characteristics of participants with or without missing covariates are shown in S1 Table.

During the follow-up period, 22 (1.3%) participants relocated from Minami-Aizu Town or Tadami Town without LOI. The median follow-up duration was 5.81 years (IQR: 5.74–5.85 years). Table 1 shows the baseline characteristics. The median age was 71 years (IQR: 68–75 years) and 40.5% of the participants were men. The mean SOC score was 17.2 (standard deviation [SD], 4.2). The median SOC score for each SOC category is presented in Table 1.

**Table 1:** Participants’ baseline characteristics.

	Total	High SOC	Lower SOC	Lowest SOC	p value
	N = 1,661	N = 1,309	N = 224	N = 128	
Age [year], median (IQR)	71 (68-75)	72 (68-75)	71 (67-75)	72 (69-74)	0.28
Male, n (%)	672 (40.5%)	536 (40.9%)	87 (38.8%)	49 (38.3%)	0.73
Body Mass Index (BMI), median (IQR)	23.7 (21.6-25.6)	23.7 (21.6-25.6)	23.9 (21.4-25.7)	23.8 (21.1-25.5)	0.94
Living alone, n (%)	178 (10.7%)	146 (11.2%)	19 (8.5%)	13 (10.2%)	0.48
Marital status, n (%)					0.21
Never	29 (1.8%)	23 (1.8%)	2 (0.9%)	4 (3.2%)	
Married	1,257 (77.4%)	997 (77.8%)	172 (78.5%)	88 (71.0%)	
Divorced	19 (1.2%)	15 (1.2%)	3 (1.4%)	1 (0.8%)	
Widowed	302 (18.6%)	236 (18.4%)	39 (17.8%)	27 (21.8%)	
Other	17 (1.0%)	10 (0.8%)	3 (1.4%)	4 (3.2%)	
Employment status, n (%)					0.053
Current job	299 (18.0%)	250 (19.1%)	37 (16.5%)	12 (9.4%)	
Part-time job	93 (5.6%)	75 (5.7%)	9 (4.0%)	9 (7.0%)	
Other	1,269 (76.4%)	984 (75.2%)	178 (79.5%)	107 (83.6%)	
Current smoker, n (%)	152 (9.2%)	122 (9.3%)	21 (9.4%)	9 (7.0%)	0.69
Alcohol (never/ < 20g/>=20g)	647/760/254	494/600/215	90/106/28	63/54/11	0.033
Number of comorbidities (0/1/2-)	655/683/323	516/544/249	94/88/42	45/51/32	0.48
Hypertension	810 (48.8%)	632 (48.3%)	110 (49.1%)	68 (53.1%)	0.57
Diabetes mellitus	123 (7.4%)	96 (7.3%)	15 (6.7%)	12 (9.4%)	0.64
Kidney disease	49 (3.0%)	33 (2.5%)	11 (4.9%)	5 (3.9%)	0.12
Cerebrovascular disease	89 (5.4%)	68 (5.2%)	10 (4.5%)	11 (8.6%)	0.22
Respiratory disease	76 (4.6%)	67 (5.1%)	6 (2.7%)	3 (2.3%)	0.12
Cardiovascular disease	157 (9.5%)	125 (9.5%)	19 (8.5%)	13 (10.2%)	0.85
Hepatic disease	41 (2.5%)	31 (2.4%)	7 (3.1%)	3 (2.3%)	0.79
Anemia	63 (3.8%)	49 (3.7%)	6 (2.7%)	8 (6.3%)	0.24
MHI-5 total score, median (IQR)	80 (65-95)	85 (65-95)	75 (60-90)	70 (60-90)	<0.001
IPAQ (METs/week), median (IQR)	6720 (2232-14532)	7038 (2433-14598)	6000 (2093-13518)	4968 (1089-14167)	0.028
SOC, median (IQR)	18 (15-21)	20 (17-21)	12 (12-14)	8 (5-10)	<0.001

Statistical differences between participants are presented. Continuous variables are compared using the Kruskal-Wallis test, and categorical variables are compared using Pearson’s chi-square test. P values are provided for these comparisons.

The SOC score was converted into a standardized score using the national mean SOC score from a previous national representative survey [36]. The standardized score was then categorized into three groups: the lowest SOC group (standardized score lower than 40), lower SOC group (standardized score ranging from 40 to 49), and high SOC group (standardized score ranging from 50 or higher, that is, equal to or exceeding the national mean score).

Missing data: marital status (n = 37). IQR denotes interquartile range. MHI-5 denotes Mental Health Inventory-5. SOC denotes sense of coherence.

### Association of SOC with LOI or death

The overall incidence of LOI or death was 128/1,661 (7.7%): LOI was 54/1,661 (3.3%) and death without LOI was 74/1,661 (4.5%). Of the 54 participants with LOI, 19 had severe dementia. We found that a lower SOC was associated with an increased risk of LOI or death (Table 2). The crude HR was 1.27 (95% CI: 1.09–1.48, *p* = 0.002) per 1 SD decrease in the SOC score. Furthermore, multivariable analysis showed a similar association between a lower SOC and a higher risk of LOI or death. The multivariable-adjusted HR was 1.25 (95% CI: 1.07–1.46, *p* = 0.005) per 1 SD decrease in the SOC score.

**Table 2:** Association of SOC with LOI or death.

	Cumulative	Crude		Age and sex adjusted		Multivariable adjusted*	
	Incidence	HR (95%CI)	p value	HR (95%CI)	p value	HR (95%CI)	p value
***Continuous variable***							
SOC (per 1 SD decrease)	128/1,661 (7.7%)	1.27 (1.09–1.48)	0.002	1.31 (1.12–1.52)	0.001	1.25 (1.07–1.46)	0.005
***Categorical variable***							
High SOC	87/1,309 (6.6%)	reference	-	reference	-	reference	-
Lower SOC	20/224 (8.9%)	1.34 (0.83–2.19)	0.23	1.48 (0.91–2.40)	0.12	1.39 (0.85–2.27)	0.19
Lowest SOC	21/128 (16.4%)	2.62 (1.62–4.23)	<0.001	2.88 (1.79–4.65)	<0.001	2.46 (1.51–4.02)	<0.001

Abbreviations: HR, hazard ratio; CI, confidence interval; SD, standard deviation.

We used Cox proportional hazard model.

We treated SOC-3-UTHS as a continuous variable and calculated the HR per 1 SD decrease.

Subsequently, we converted SOC-3-UTHS based on national reference value and categorized them as lowest SOC (below 40), lower SOC (40 to 49), and high SOC (50 or higher).

*Adjusted for age, sex, body mass index, living alone or not, employment status, number of comorbidities, smoking, alcohol intake, mental health and physical activity.

In the sensitivity analysis, the estimate was similar after additional adjustment for marital status and the Social Functioning score of the SF-36 (adjusted HR, 1.27 [95% CI, 1.08–1.49]; *p* = 0.004). Furthermore, when SOC was treated as a categorical variable, we observed a similar association between lower SOC and an increased risk of LOI or death. Compared with the high SOC group, the multivariable-adjusted HR was 1.39 (95% CI, 0.85–2.27; *p* = 0.19) in the lower SOC group and 2.46 (95% CI, 1.51–4.02; *p* < 0.001) in the lowest SOC group (Table 2). Finally, the sensitivity analysis using imputed datasets yielded conclusions similar to those of the main analysis regarding the association between SOC and LOI or death. The age- and sex-adjusted HR per 1-SD decrease in SOC-3-UTHS was 1.23 (95% CI, 1.07–1.41; *p* = 0.003), and the multivariable-adjusted HR was 1.17 (95% CI, 1.02–1.35; *p* = 0.029).

### Association of SOC with death

The overall incidence of death was 94/1,661 (5.7%). We found that a lower SOC was associated with a higher risk of death (Table 3). The multivariable-adjusted HR was 1.24 (95% CI: 1.03–1.50, *p* = 0.022) per 1 SD decrease in the SOC score. In the sensitivity analysis, where SOC was treated as a categorical variable, the multivariable-adjusted HR was 1.49 (95% CI: 0.84–2.64, *p* = 0.17) in the lower SOC group and 2.17 (95% CI: 1.19–3.96, *p* = 0.011) in the lowest SOC group, both compared to the high SOC group.

**Table 3:** Association of SOC with death.

	Cumulative	Crude		Age and sex adjusted		Multivariable adjusted*	
	Incidence	HR (95%CI)	p value	HR (95%CI)	p value	HR (95%CI)	p value
***Continuous variable***							
SOC (per 1 SD decrease)	94/1,661 (5.7%)	1.24 (1.04–1.48)	0.019	1.28 (1.08–1.53)	0.006	1.24 (1.03–1.50)	0.022
***Categorical variable***							
High SOC	65/1,309 (5.0%)	reference	-	reference	-	reference	-
Lower SOC	15/224 (6.7%)	1.35 (0.77–2.37)	0.29	1.53 (0.87–2.68)	0.14	1.49 (0.84–2.64)	0.17
Lowest SOC	14/128 (10.9%)	2.25 (1.26–4.00)	0.006	2.48 (1.39–4.43)	0.002	2.17 (1.19–3.96)	0.011

Abbreviations: HR, hazard ratio; CI, confidence interval; SD, standard deviation.

We used Cox proportional hazard model.

We treated SOC-3-UTHS as a continuous variable and calculated the HR per 1 SD decrease.

Subsequently, we converted SOC-3-UTHS based on national reference value and categorized them as lowest SOC (below 40), lower SOC (40 to 49), and high SOC (50 or higher).

*Adjusted for age, sex, body mass index, living alone or not, employment status, number of comorbidities, smoking, alcohol intake, mental health and physical activity.

We also assessed the association between SOC and cause-specific mortality. Cancer was the leading cause of death in this cohort, followed by cardiovascular diseases (Table 4). The multivariable-adjusted HR for cancer death was 1.23 (95% CI: 0.92–1.64, *p* = 0.16) per 1 SD decrease in the SOC score. The multivariable-adjusted HR for cardiovascular death was 1.61 (95% CI: 1.16–2.23, *p* = 0.005) per 1 SD decrease in the SOC score. Other causes of death included external causes (n = 10), respiratory diseases (n = 7), neurological diseases (n = 4), renal diseases (n = 2), senility (n = 2), infectious diseases (n = 1), and gastrointestinal diseases (n = 1).

**Table 4:** Association of SOC with cause-specific death.

	Cumulative	Crude		Age and sex adjusted		Multivariable adjusted*	
	Incidence	HR (95%CI)	p value	HR (95%CI)	p value	HR (95%CI)	p value
***Cancer death***							
SOC (per 1 SD decrease)	40/1,661 (2.4%)	1.21 (0.91–1.60)	0.19	1.25 (0.94–1.64)	0.12	1.23 (0.92–1.64)	0.16
***Cardiovascular death***							
SOC (per 1 SD decrease)	27/1,661 (1.6%)	1.55 (1.14–2.09)	0.004	1.62 (1.21–2.18)	0.001	1.61 (1.16–2.23)	0.005

Abbreviations: HR, hazard ratio; CI, confidence interval; SD, standard deviation.

We used Cox proportional hazard model.

We treated SOC-3-UTHS as a continuous variable and calculated the HR per 1 SD decrease.

*Adjusted for age, sex, body mass index, living alone or not, employment status, number of comorbidities, smoking, alcohol intake, mental health and physical activity.

## Discussion

In this study, a lower SOC was associated with a higher incidence of LOI or death in community-dwelling older adults, even after adjusting for potential confounders. These results were consistent when SOC was treated as a continuous or categorical variable. We also found that a lower SOC was associated with a higher incidence of death.

Our results are consistent with those of previous studies regarding the association between SOC and LOI or death in older adults. One study with adults aged 80 years or over indicated that individuals with a higher SOC tended to show less functional decline and mortality [12]. However, this study included fewer than 500 patients in a primary care setting, which led to broad CIs for evaluating functional decline. Furthermore, the follow-up duration for functional decline in the study was relatively short, averaging 19.6 months ± 2.5 months. Another study showed that a higher SOC was associated with lower disability in patients with low-back pain surgery in 5 years of observation [39]. However, individuals in the study were limited to those who underwent low-back pain surgery. Our finding extends these results to a large general population. Regarding mortality, previous studies have also demonstrated that lower SOC is associated with an increased risk of mortality [40–46]. Our study further evaluated cause-specific mortality and LOI or death.

We lacked information regarding the precise cause of LOI. However, 19 of the 54 participants who experienced LOI were assessed as having severe dementia based on the level of independence in daily living for patients with dementia. In addition, a national survey indicated that dementia is the most common cause of LTCI certification status, which leads to LOI [47]; thus, cognitive decline may partially account for instances of LOI. Furthermore, a recent study showed an association between SOC and subsequent onset of dementia [32]. According to the abovementioned national survey, the primary reasons for the LTCI certification status, aside from dementia, are stroke and fracture/falls, followed closely by frailty [47]. SOC was linked to the incidence of stroke in a longitudinal study [18] and frailty in a cross-sectional study [48]. Therefore, we hypothesized that SOC may be associated with various causes of LOI, including dementia, stroke, fracture/falls, and frailty. Further research is required to elucidate the mechanisms linking SOC and LOI.

We found that a lower SOC was associated with an increased risk of cardiovascular mortality, and we observed a trend of association with cancer mortality, although not statistically significant. The link between SOC and cardiovascular diseases or cancer has been documented in previous studies [17,49]. In cardiovascular diseases, higher SOC has been associated with higher adherence to hypertension medication [14]. Moreover, higher SOC has been linked to the maintenance of physical activity [50]. Thus, the positive effects of medication adherence and physical activity on health could explain the association between SOC and cardiovascular diseases. A previous study found that older individuals with a higher SOC were less likely to develop cancer [49]. Several underlying factors could influence this; previous studies have suggested that individuals with a high SOC may be less likely to engage in high-risk behaviors, such as smoking and excessive alcohol consumption [51,52]. Our observations provide avenues for more focused research on the mechanisms linking SOC with cause-specific mortality, including cardiovascular diseases and cancer.

Several potential mechanisms may link SOC to LOI or death. First, regarding comprehensibility, people who can predict future events might adopt preventive strategies such as vaccine uptake [53]. Additionally, those with a high SOC understand the significance of treatment; for instance, individuals with a high SOC showed greater adherence to secondary prevention strategies following myocardial infarction [54]. Second, regarding manageability, individuals capable of handling stressors often employ more adaptive coping styles, such as active coping, positive reframing, or seeking support, rather than maladaptive strategies such as catastrophic thoughts, self-blame, or substance use [11,55,56]. Notably, seeking social support has emerged as a crucial strategy for managing frailty [57]. Third, regarding meaningfulness, those who find significance in life events tend to maintain a healthy mental state even in the face of negative life events, which may protect them against depression [11] and dementia [32]. Indeed, SOC is strongly associated with mental health status [58] and SOC appears to reflect resilience to stressful life events [42,59], contributing to prolonged disability-free survival [12].

Our results suggest that SOC is a promising target for improving disability-free survival and promoting healthy aging. Childhood environments, such as lower socioeconomic status and limited social support, have been suggested to contribute to lower SOC in adolescence [60], and SOC has been considered relatively stable throughout adulthood [6]. However, recent studies have suggested that SOC can be modifiable [44,61]. A randomized controlled trial revealed that an empowerment program over two months led to an improved SOC in older adults with at least one chronic disease [9]. Similarly, another randomized controlled trial showed that a three-month self-care program consisting of 24 activities enhanced older adults’ SOC [10]. These results suggest that SOC can be enhanced even in older adults. Further studies are needed to investigate whether these approaches to increase SOC would prevent severe disability.

The strength of this study is that it is a population-based cohort study including community-dwelling older adults, longitudinal design, and a high follow-up rate and measuring clinically important outcomes for older adults (i.e., LOI or death). This study also has limitations. First, although we adjusted for key covariates identified in previous studies, including age, sex, comorbidities, employment status, smoking status, and alcohol consumption [40,43], and obtained consistent findings in sensitivity analyses additionally adjusting for marital status and the Social Functioning domain of the SF-36, we could not directly measure socioeconomic status such as educational attainment and financial circumstances. Therefore, residual confounding by these unmeasured factors cannot be excluded. Second, although we assessed the prevalence of severe dementia in these cases, we did not have information regarding the exact causes of LOI. Third, in this study, SOC was assessed using a three-item scale, although the original scale consisted of 29 items (SOC-29) [8,58]. However, the validity of the three-item SOC scale has been confirmed in a previous study [25]. Fourth, there were missing data in the covariates, and participant characteristics differed between those with and without missing data (S1 Table). Specifically, those with missing data were older, less likely to have a current full-time job, and more likely to be widowed. Therefore, we cannot exclude the possibility that this may have affected the study results. Fifth, this study was conducted in only two rural towns in Japan, and it is unclear whether our results can be applied to other regions. The mean SOC score in this study was higher than that reported for the general population in Japan [36]. The study participants may have been more health-conscious than the general population, warranting caution when generalizing the results to other populations. Finally, SOC was measured only once at baseline, and changes in SOC during follow-up could not be assessed. Although SOC is considered a relatively stable orientation and we adjusted for baseline mental health using the MHI-5, the possible influence of emotional status due to major life events cannot be excluded [27,61].

In this population-based cohort study, we found that a lower SOC was associated with an increased risk of LOI or death in community-dwelling older adults. Enhancing SOC may be important for reducing LOI or death and promoting healthy aging in older adults.

## Supporting information

S1 Table Participants’ baseline characteristics with or without missing covariates. (DOCX)
