# Supplementary material for: Association between sense of coherence and loss of independence or death in older adults: Locomotive Syndrome and Health Outcomes in Aizu Cohort Study
Source: PLoS One. 2026 Aug 31;21(8):e0355726. doi: 10.1371/journal.pone.0355726 (PMC13528987; doi:10.1371/journal.pone.0355726)
Supplement: Supplementary file 1 [file pone.0355726.s001.docx]

| **S1 Table.**  Participants’ baseline characteristics with or without missing covariates | | |  |
| --- | --- | --- | --- |
|  | Missing  Covariates (-) | Missing  Covariates (+) |  |
|  | N= 1,661 | N=466 | p value |
| Age [year], median (IQR) | 71 (68-75) | 73 (69-77) | <0.001 |
| Male, n (%) | 672 (40.5%) | 180 (38.6%) | 0.48 |
| Body Mass Index (BMI), median (IQR) | 23.7  (21.6-25.6) | 23.7  (21.3-25.6) | 0.32 |
| Living alone, n (%) | 178 (10.7%) | 44 (10.0%) | 0.64 |
| Marital status, n (%) |  |  | <0.001 |
| Never | 29 (1.8%) | 7 (1.6%) |  |
| Married | 1,257 (77.4%) | 315 (70.8%) |  |
| Divorced | 19 (1.2%) | 1 (0.2%) |  |
| Widowed | 302 (18.6%) | 120 (27.0%) |  |
| Other | 17 (1.0%) | 2 (0.4%) |  |
| Employment status, n (%) |  |  | 0.012 |
| Current job | 299 (18.0%) | 61 (14.0%) |  |
| Part-time job | 93 (5.6%) | 14 (3.2%) |  |
| Other | 1,269 (76.4%) | 360 (82.8%) |  |
| Current smoker, n (%) | 152 (9.2%) | 19 (10.5%) | 0.87 |
| Alcohol (never/<20g/>=20g) | 647/760/254 | 96/121/36 | 0.81 |
| Number of comorbidities (0/1/2-) | 655/683/323 | 81/109/46 | 0.26 |
| Hypertension | 810 (48.8%) | 134 (55.1%) | 0.063 |
| Diabetes mellitus | 123 (7.4%) | 19 (7.9%) | 0.76 |
| Kidney disease | 49 (3.0%) | 8 (3.3%) | 0.75 |
| Cerebrovascular disease | 89 (5.4%) | 11 (4.6%) | 0.62 |
| Respiratory disease | 76 (4.6%) | 16 (6.1%) | 0.28 |
| Cardiovascular disease | 157 (9.5%) | 21 (8.5%) | 0.63 |
| Hepatic disease | 41 (2.5%) | 7 (2.9%) | 0.68 |
| Anemia | 63 (3.8%) | 8 (3.3%) | 0.73 |
| MHI-5 total score, median (IQR) | 80 (65-95) | 80 (60-90) | 0.004 |
| IPAQ (METs/week), median (IQR) | 6,720  (2,232-14,532) | 6,533  (2,242-13,313) | 0.50 |
| SOC, median (IQR) | 18 (15-21) | 18 (15-21) | 0.20 |
| IQR denotes interquartile range. | | | |
| MHI-5 denotes Mental Health Inventory-5. | | | |
| IPAQ denotes International Physical Activity Questionnaire. | | | |
| SOC denotes sense of coherence. | | | |
| Statistical differences between participants with and without missing covariates are presented. Continuous variables are compared using the Wilcoxon rank-sum test, and categorical variables are compared using Pearson’s chi-square test. P values are provided for these comparisons. | | | |
