# Supplementary material for: Association between sense of coherence and loss of independence or death in older adults: Locomotive Syndrome and Health Outcomes in Aizu Cohort Study
Source: PLoS One. 2026 Aug 31;21(8):e0355726. doi: 10.1371/journal.pone.0355726 (PMC13528987; doi:10.1371/journal.pone.0355726)
Supplement: Supplementary file 4 [file pone.0355726.s005.docx]

July 22, 2026

**Dear editor,**

Thank you for your constructive comments. We appreciate the opportunity to resubmit our manuscript entitled, “Association between sense of coherence and loss of independence or death in older adults: Locomotive Syndrome and Health Outcomes in Aizu Cohort Study,” for publication in *PLOS One* as an original article. We have revised our paper in accordance with all the comments and helpful suggestions made by the editors and reviewers.

In accordance with your instructions, we have prepared the following files:

1) Point-by-point responses

2) Revised manuscript with track change to show the edits

3) Revised clean copy of our manuscript, figures, and supplementary materials

**POINT-BY-POINT RESPONSES TO EDITORS’ AND REVIEWERS’ COMMENTS:**

**Journal Requirements:
If the reviewer comments include a recommendation to cite specific previously published works, please review and evaluate these publications to determine whether they are relevant and should be cited. There is no requirement to cite these works unless the editor has indicated otherwise.

Please review your reference list to ensure that it is complete and correct. If you have cited papers that have been retracted, please include the rationale for doing so in the manuscript text, or remove these references and replace them with relevant current references. Any changes to the reference list should be mentioned in the rebuttal letter that accompanies your revised manuscript. If you need to cite a retracted article, indicate the article’s retracted status in the References list and also include a citation and full reference for the retraction notice.**

Response:

Thank you for this comment. We carefully reviewed the reference list to ensure that all citations are accurate, complete, and up to date. We also confirmed that none of the cited references have been retracted. In accordance with the Editor’s request, we added one reference providing evidence for the temporal stability of the sense of coherence (SOC) measure in a Japanese population. This change has been incorporated into both the manuscript and the reference list. No other changes to the reference list were necessary

**Additional Editor Comments:**

**Dear Authors,**

**Thank you for submitting the revised manuscript and the detailed point-by-point response. The reviewers’ evaluations differed in the previous round, with one reviewer recommending acceptance and the other raising remaining concerns. I have reviewed your responses to these remaining concerns and consider that most of the issues have been adequately addressed.**

**Before proceeding to a final decision, please make a minor editorial revision regarding the description of sense of coherence. In your response letter, you provided evidence that SOC is relatively stable over time, including findings from a Japanese panel study. However, the corresponding text added to the manuscript is rather brief. Please incorporate this supporting evidence more explicitly into the manuscript, while also retaining the limitation that baseline SOC may still be influenced by emotional status or major life events.**

**No further external review is required at this stage. Please submit a clean revised manuscript and a brief response indicating where this change has been made.**

**I look forward to receiving your revised manuscript.**

Response:

Thank you for your helpful suggestion. We have expanded the description of the temporal stability of SOC in the Methods section (lines 117–120, page 8 of the revised manuscript) by adding evidence from a three-wave Japanese panel study, including the invariance of the factor structure of the SOC3-UTHS across time and stability coefficients of 0.69 and 0.70 between consecutive annual assessments. We have also retained the limitation in the Discussion (lines 334–337, page 25 of the revised manuscript) that baseline SOC may have been influenced by participants’ emotional status or major life events.

We feel that the revision has improved our manuscript and hope that it is now suitable for publication.

Sincerely,

**Yosuke Yamamoto, MD, PhD**

Department of Healthcare Epidemiology, Graduate School of Medicine, Kyoto University

54 Kawahara-cho, Syogoin, Sakyo-ku, Kyoto 606-8501, Japan

E-mail: [yamamoto.yosuke.5n@kyoto-u.ac.jp](mailto:yamamoto.yosuke.5n@kyoto-u.ac.jp)
