# Supplementary material for: Association between sense of coherence and loss of independence or death in older adults: Locomotive Syndrome and Health Outcomes in Aizu Cohort Study
Source: PLoS One. 2026 Aug 31;21(8):e0355726. doi: 10.1371/journal.pone.0355726 (PMC13528987; doi:10.1371/journal.pone.0355726)
Supplement: Supplementary file 5 [file pone.0355726.s003.docx]

May 24, 2026

**Dear editors and reviewers**,

Thank you for your constructive comments and important suggestions. We appreciate the opportunity to resubmit our manuscript entitled, “Association between sense of coherence and loss of independence or death in older adults: Locomotive Syndrome and Health Outcomes in Aizu Cohort Study,” for publication in *PLOS One* as an original article. We have revised our paper in accordance with all the comments and helpful suggestions made by the editors and reviewers.

In accordance with your instructions, we have prepared the following files:

1) Point-by-point responses

2) Revised manuscript with track change to show the edits

3) Revised clean copy of our manuscript, figures, and supplementary materials

We feel that the revision has improved our manuscript and hope that it is now suitable for publication.

Kind regards,

**Yosuke Yamamoto, MD, PhD**

Department of Healthcare Epidemiology, Graduate School of Medicine, Kyoto University

54 Kawahara-cho, Syogoin, Sakyo-ku, Kyoto 606-8501, Japan

E-mail: [yamamoto.yosuke.5n@kyoto-u.ac.jp](mailto:yamamoto.yosuke.5n@kyoto-u.ac.jp)

**POINT-BY-POINT RESPONSES TO EDITORS’ AND REVIEWERS’ COMMENTS:**

**Editor:**

**Dear Authors,**

**Thank you for submitting your manuscript examining the association between sense of coherence (SOC) and loss of independence or death among older adults. The study addresses an important research question and is supported by a well-defined cohort and longitudinal design. The reviewers found the topic to be relevant and the analysis generally appropriate.**

**However, after careful evaluation of the manuscript and reviewer comments, several issues require substantial clarification and improvement before the manuscript can be considered further. These primarily concern the reporting of key variables, handling of confounding (particularly social factors), and interpretability of the findings.**

**A major revision is therefore required.**

Response:

Thank you for your interest in our work and for your helpful comments. We have revised the manuscript and responded point-by-point.

**1. Reporting of Exposure (SOC)**

**The reporting of the primary exposure is currently insufficient.**

**Please provide descriptive statistics for SOC, including:**

**- Mean and distribution**

**- Categorized presentation (e.g., tertiles or predefined groups)**

**- Participant characteristics by SOC category**

Response:

Thank you for your comment regarding the reporting of SOC. We revised the manuscript to address each of these points regarding the presentation of SOC.

- Mean and distribution

We added descriptive statistics of SOC in the Results section, including the mean and standard deviation:

“The mean SOC score was 17.2 (standard deviation [SD], 4.2). The median SOC score for each SOC category is presented in Table 1.” (lines 196–198, pages 12-13 of the revised manuscript).

- Categorized presentation

SOC was categorized into three groups based on standardized scores for descriptive and comparative analyses, and the median and interquartile ranges across these SOC categories were added to Table 1.

- Participant characteristics by SOC category

We revised Table 1 to present participant characteristics stratified by SOC category.

**Please clarify the measurement of SOC (SOC3-UTHS), including:**

**- Brief description of items (full wording optional if previously validated and cited)**

**- Evidence on reliability and validity (with quantitative indicators if available)**

Response:

Thank you for your thoughtful comments regarding the measurement of SOC. We have revised the manuscript to address each of these points.

- Brief description of items

We clarified the measurement of SOC by adding a brief description of the SOC-3-UTHS items in the Methods section:

“The SOC-3-UTHS is a Japanese short version of the SOC scale, comprising three items that address manageability (“I am able to find solutions to the hardships and problems that occur every day”), meaningfulness (“I think it is worth facing and dealing with some of the hardships and problems of life”), and comprehensibility (“I am able to understand and predict the hardships and problems that occur every day”) [26].” (lines 109–113, pages 7–8 of the revised manuscript).

- Evidence on reliability and validity

We added information regarding the psychometric properties of the SOC-3-UTHS, including previously reported reliability and validity indices:

“The validity and reliability of SOC-3-UTHS have been shown in a previous study, reporting good internal consistency (Cronbach’s α = 0.84) and a moderate correlation with the SOC-13 (r = 0.51) [25].” (lines 115–117, page 8 of the revised manuscript)

**2. Confounding and Interpretation (Critical Issue)**

**The interpretation of the association between SOC and outcomes requires further strengthening.**

**The potential for residual confounding by socioeconomic and social factors needs more explicit treatment:**

**- Clarify whether SES variables were available**

**- If unavailable, discuss this more explicitly as a limitation**

Response:

Thank you for highlighting the important issue of residual confounding by socioeconomic and social factors. We clarified that data on educational attainment and income were not available in our dataset, and we explicitly acknowledged this as a limitation of the study. As proxies for socioeconomic and social factors, we used marital status and the Social Functioning domain of the 36-Item Short Form Health Survey (SF-36), and additionally adjusted for these variables in a sensitivity analysis. The estimate was similar after additional adjustment (adjusted HR, 1.27 [95% CI, 1.08–1.49]; p = 0.004).

“First, we additionally adjusted for marital status and the Social Functioning score of the 36-Item Short Form Health Survey (SF-36) to account for social factors in the relationship between SOC and the outcome [34].” (lines 158-160, page 10 of the revised manuscript)

“In the sensitivity analysis, the estimate was similar after additional adjustment for marital status and the Social Functioning score of the SF-36 (adjusted HR, 1.27 [95% CI, 1.08–1.49]; p = 0.004).” (lines 212-214, page 17 of the revised manuscript)

“First, we could not adjust for socioeconomic status such as educational attainment and income, which may have led to residual confounding. However, we adjusted for the most important covariates considered in previous studies (age, sex, comorbidities, employment status, smoking status, and alcohol consumption) [39,42]. Moreover, the sensitivity analyses additionally adjusting for marital status and the Social Functioning domain of the SF-36 showed consistent findings.” (lines 313-318, pages 24-25 of the revised manuscript)

**- Expand discussion on the relationship between SOC and SES based on prior literature**

Response:

Thank you for this important suggestion. We expanded our discussion regarding the relationship between SOC and socioeconomic status (SES) based on prior literature. Childhood environments, such as lower SES have been suggested to contribute to lower SOC in adolescence (Soc Psychiatry Psychiatr Epidemiol. 2009 Dec;44(12):1097-105.). On the other hand, higher SOC has been suggested to help gain social relationships (The Handbook of Salutogenesis [Internet]. 2nd ed. Cham (CH): Springer. 2022). Therefore, it was difficult to define SES as confounder or mediators. We explicitly discussed this point in the Method and Discussion section.

“First, we additionally adjusted for marital status and the Social Functioning score of the 36-Item Short Form Health Survey (SF-36) to account for social factors in the relationship between SOC and the outcome [34]. These variables were not included in the primary analysis because social factors may act not only as confounders but also as potential mediators in the relationship between SOC and health outcomes, given the potentially bidirectional relationship between SOC and social factors [8].” (lines 158-163, pages 10-11 of the revised manuscript)

“Childhood environments, such as lower socioeconomic status and limited social support, have been suggested to contribute to lower SOC in adolescence [59], and SOC has been considered relatively stable throughout adulthood. However, recent studies have suggested that SOC can be modifiable [43, 60].” (lines 300-304, page 24 of the revised manuscript)

**Similarly, please address the potential role of social participation or community engagement:**

**- If data are available, consider additional adjustment**

**- If not, acknowledge this as a limitation**

Response:

Thank you for this valuable suggestion. While we could not directly measure “social participation” or “community engagement,” we had data regarding the Social Functioning domain of SF-36 (Med Care. 1992;30:473–483.) We conducted an additional sensitivity analysis further adjusting for marital status and the Social Functioning domain of the SF-36, and the results remained consistent.

“First, we additionally adjusted for marital status and the Social Functioning score of the 36-Item Short Form Health Survey (SF-36) to account for social factors in the relationship between SOC and the outcome [34].” (lines 158-160, page 10 of the revised manuscript)

“In the sensitivity analysis, the estimate was similar after additional adjustment for marital status and the Social Functioning score of the SF-36 (adjusted HR, 1.27 [95% CI, 1.08–1.49]; p = 0.004).” (lines 212-214, page 17 of the revised manuscript)

We also added the limitation that community engagement was not measured in the revised manuscript.

“First, we could not adjust for community engagement and socioeconomic status such as educational attainment and income, which may have led to residual confounding.” (lines 313-315, page 24 of the revised manuscript)

**3. Missing Data and Sample Selection
- Please report statistical comparisons between participants included and excluded due to missing data.
- Provide SOC values for these groups if possible.**

Response:

Thank you for highlighting the importance of assessing potential bias due to missing data.

- Please report statistical comparisons between participants included and excluded due to missing data.

We added statistical comparisons between participants included in the analysis and those excluded due to missing data, which are presented in S1 Table.

- Provide SOC values for these groups if possible.

We have provided SOC values for both included and excluded participants in S1 Table.

**- Ensure that findings from supplementary analyses (e.g., S1 Table) are incorporated into the Discussion where relevant.**

We have incorporated the findings from S1 Table into the Discussion section. Participants with missing data were older, less likely to have a current full-time job, and more likely to be widowed. We have discussed how these differences may have influenced the observed associations.

“Fourth, there were missing data in the covariates, and participant characteristics differed between those with and without missing data (S1 Table). Specifically, those with missing data were older, less likely to have a current full-time job, and more likely to be widowed. Therefore, we cannot exclude the possibility that this may have affected the study results.” (lines 322-326, page 25 of the revised manuscript)”

**4. Study Description and Transparency**

**Provide key cohort information within the manuscript (even if previously published), including:**

**- Recruitment process**

**- Follow-up duration**

**- Key inclusion/exclusion criteria**

Response:

Thank you for your helpful suggestions to improve the clarity and completeness of the cohort description. We have provided the key cohort information.

- Recruitment process

We added details regarding participant recruitment process.

“The study recruited community-dwelling participants aged 40–80 years who were National Health Insurance beneficiaries and participated in annual health checkups conducted by their local governments between 2008 and 2010 [23].”(lines 82–85, page 6 of the revised manuscript)

- Follow-up duration

We clarified the prospective follow-up process and current study status.

“After the baseline examination, participants were followed prospectively, and the study is currently ongoing.” (lines 91–92, page 6 of the revised manuscript)

- Key inclusion/exclusion criteria

We added key information regarding the study population.

“Survey forms were distributed to individuals attending the health checkups, and those who agreed to participate in the study returned the forms (the response rate was 60% in the first year) [23]. Approximately 5,800 individuals were recruited between 2008 and 2010 [24]. The baseline survey consisted of self-administered questionnaires, health checkups including blood and urine tests, musculoskeletal examinations, assessments of motor dysfunction, and radiographic assessments [23].” (lines 85–91, page 6 of the revised manuscript)

**Expand the description of the study setting (Minami-Aizu and Tadami) to aid interpretation of generalizability.**

Response:

We have expanded the description of the study setting, including characteristics of Minami-Aizu and Tadami, to provide better context for interpreting the generalizability of our findings.

“Tadami Town and Minami-Aizu Town are rural, mountainous regions, and the majority of participants were aged 70 years or older.” (lines 79-81, page 6 of the revised manuscript)

**In Figure 1, include the number of participants censored due to relocation.**

Response:

Thank you for pointing this out. We have revised Figure 1 to include the number of participants censored due to relocation. The number of participants lost to follow-up was 22, and the remaining 1,639 participants completed the follow-up.

**5. Clarification of Analytical Choices
Provide clearer justification for:
- The treatment of BMI as a sensitivity analysis
- The categorization of employment status**

Response:

Thank you for these important comments regarding our analytical choices. Following the reviewers’ suggestions, we revised the primary model by including BMI and modifying the categorization of employment status. The results remained statistically significant.

- The treatment of BMI as a sensitivity analysis

In the original analysis, BMI was included as part of a sensitivity analysis due to the controversy in relationship with SOC. However, following the reviewers’ suggestions, we reconsidered this approach and have now included BMI as a covariate in the primary analysis. The multivariable-adjusted HR was 1.25 (95% CI: 1.07–1.46, *p* = 0.005) per 1 SD decrease in the SOC score, which was consistent with the original findings.

- The categorization of employment status

Employment status was initially dichotomized as “full-time” versus “not full-time” to reflect the relatively high proportion of participants engaged in full-time work. However, to better capture heterogeneity in employment, we have revised the categorization to “full-time,” “part-time,” and “other.” The multivariable-adjusted HR was 1.25 (95% CI: 1.07–1.46, *p* = 0.005) per 1 SD decrease in the SOC score, which was consistent with the original findings.

“As potential confounders, we adjusted for age, sex, body mass index, employment status (having a current full-time job, having a part-time job or other), living alone or not, number of comorbidities, alcohol consumption (none, less than 20 g/day, 20 g/day or more), smoking status (current smoker or not), baseline mental health status, and baseline physical activity.” (lines 139-142, page 9 of the revised manuscript)

“Furthermore, multivariable analysis showed a similar association between a lower SOC and a higher risk of LOI or death. The multivariable-adjusted HR was 1.25 (95% CI: 1.07–1.46, *p* = 0.005) per 1 SD decrease in the SOC score. (lines 206-208, pages 14-15 of the revised manuscript)

**Clarify which covariates are referred to as “important covariates in previous studies.”**

Response:
Thank you for raising this point. We have clarified which covariates were considered important based on prior literature by explicitly listing them in the Discussion section.

“However, we adjusted for the most important covariates considered in previous studies (age, sex, comorbidities, employment status, smoking status, and alcohol consumption) [39, 42].” (lines 315-317, page 24 of the revised manuscript)

**6. Interpretation and Discussion
Ensure that statements regarding behavioral mechanisms (e.g., smoking, alcohol use) are:
- Supported by your data, or
- Clearly referenced as prior evidence**

Response:

Thank you for the constructive comments regarding the interpretation of our results. In our dataset, SOC was not associated with alcohol use or smoking. One possible explanation for the lack of association in our study is a limitation of the questionnaire, as excessive smoking or hazardous alcohol consumption may not have been fully captured.

Previous studies have suggested that SOC may be related to health-related behaviors such as smoking and alcohol consumption, but the evidence has not been entirely consistent (Cad Saude Publica. 2024 Sep 20;40(9):e00141323). Therefore, we have revised the manuscript to clarify that these behavioral mechanisms were discussed based on prior literature and modified the wording to a more cautious expression using “may.”

“Several underlying factors could influence this; previous studies have suggested that individuals with a high SOC may be less likely to engage in high-risk behaviors, such as smoking and excessive alcohol consumption [50, 51].” (lines 280-283, page 23 of the revised manuscript)

**Discuss the possibility that SOC may change over time, given that it was measured only at baseline.**

Response:

Thank you for this important comment. We agree that SOC may change over time and that this should be considered when interpreting our findings. In this study, SOC was measured only once at baseline; therefore, temporal changes in SOC during follow-up could not be assessed. Previous studies have suggested that SOC is relatively stable but may still change in response to life events, aging, or health conditions (Scandinavian Journal of Caring Sciences. 2013; 27(1):13-9.). We have now added this point to the limitations section of the revised manuscript.

“Finally, SOC was measured only once at baseline, and changes in SOC during follow-up could not be assessed.” (lines 331-332, page 25 of the revised manuscript)

**Strengthen the discussion of generalizability by comparing your findings and SOC levels with previous studies.**

Response:

Thank you for this important suggestion. In our study, the mean SOC was higher than the national standardized score suggesting that our participants were more health-conscious than general population. We have added a description noting that this may limit the generalizability of our findings.

“The mean SOC score in this study was higher than that reported for the general population in Japan [35]. The study participants may have been more health-conscious than the general population, warranting caution when generalizing the results to other populations.” (lines 328-331, page 25 of the revised manuscript)

**Summary
The manuscript has clear potential, but key aspects of reporting and interpretation require improvement. In particular, clarification of SOC measurement, handling of social confounding, and presentation of descriptive results will substantially strengthen the paper.
We encourage a careful and structured revision with a point-by-point response.**

Response:

Thank you for your interest in our work. We appreciate all the comments, which in our opinion have greatly helped us improve the paper.

**Reviewer #1:
This is a valuable study focusing on the Sense of Coherence (SOC)—a pivotal capacity for making healthy behavioral choices—and demonstrating its association with critical outcomes such as the need for long-term care and mortality. The study's significance is further bolstered by the use of a large-scale, long-term longitudinal dataset. However, certain essential information and results are currently missing. A final decision will require the following points to be addressed through clarification or supplementation.**

Response:

We are grateful to you for your valuable suggestions regarding our manuscript. Your constructive comments have allowed us to improve our paper significantly. We provide a response to each of your comments below.

**Specific Comments
P6 (Methods: Design and setting / Participants):
While previous studies are cited, please provide the minimum essential protocol information for the cohort study within this manuscript (e.g., age range of participants, total sample size, follow-up period, and recruitment methods).**

Response:

We are grateful for your valuable suggestion. In response, we have added key cohort protocol information to the manuscript, including the age range of participants, total sample size, follow-up duration, and recruitment methods, as recommended.

“The study recruited community-dwelling participants aged 40–80 years who were National Health Insurance beneficiaries and participated in annual health checkups conducted by their local governments between 2008 and 2010 [23]. Survey forms were distributed to individuals attending the health checkups, and those who agreed to participate in the study returned the forms (the response rate was 60% in the first year) [23]. Approximately 5,800 individuals were recruited between 2008 and 2010 [24]. The baseline survey consisted of self-administered questionnaires, health checkups including blood and urine tests, musculoskeletal examinations, assessments of motor dysfunction, and radiographic assessments [23]. After the baseline examination, participants were followed prospectively, and the study is currently ongoing.” (lines 82-92, page 6 of the revised manuscript)

**P7 (SOC3-UTHS):
Please include the specific items of the SOC3-UTHS in the text. Additionally, provide the reliability and validity scores for this scale.**

Response:

Thank you for your helpful comment. As you mentioned, we provided the item of SOC-3-UTHS in the main text. Additionally, we provided the reliability and validity of this scale.

“The SOC-3-UTHS is a Japanese short version of the SOC scale, comprising three items that address manageability (“I am able to find solutions to the hardships and problems that occur every day”), meaningfulness (“I think it is worth facing and dealing with some of the hardships and problems of life”) and comprehensibility (“I am able to understand and predict the hardships and problems that occur every day”) [26].” (lines 109-113, pages 7-8 of the revised manuscript)

“The validity and reliability of SOC-3-UTHS have been shown in a previous study, reporting good internal consistency (Cronbach’s α = 0.84) and a moderate correlation with the SOC-13 (r = 0.51) [25].” (lines 115-117, page 8 of the revised manuscript)

**(Note to the author: Separately, please clarify for the reviewer’s understanding—not necessarily for inclusion in the text—what criteria were used to establish the validity of the SOC3-UTHS, or its parent SOC scale. Specifically, what serves as the "gold standard" to confirm it truly measures SOC?)**

Response:

Thank you for this important comment. SOC does not have a single established “gold standard” measure because it is a theoretical psychosocial construct rather than a directly observable biological phenomenon. The original SOC concept and the SOC-29 scale were developed by Aaron Antonovsky based on qualitative interviews with individuals who had experienced severe stress and trauma, including Holocaust survivors, to understand why some people maintained good functioning despite adversity. From these observations, Antonovsky proposed that SOC consists of three interrelated components: comprehensibility, manageability, and meaningfulness.

The SOC scale was subsequently operationalized using a facet design methodology to systematically incorporate these psychological dimensions into questionnaire items. The 29-item SOC scale was originally developed, and abbreviated 13-item and 3-item versions were subsequently developed.

Because SOC is a theoretical construct, the validity of SOC scales—including the SOC-29 and abbreviated versions such as the SOC3-UTHS—has been evaluated using multiple psychometric approaches rather than comparison with a single gold standard instrument. These approaches include face validity, construct validity through factor analytic studies of the three theoretical components. The validity of these scales has also been assessed through correlations with related constructs, such as mental health and quality of life, and through longitudinal associations with health outcomes, including mortality.

The SOC3-UTHS was developed as an abbreviated measure based on the original SOC framework and has previously demonstrated acceptable psychometric properties, including correlations with established SOC measures.

**P9, L137–138:
It is unclear why a separate analysis focusing solely on BMI is necessary. Could it not be included in the primary analysis from the outset? Please explain the rationale for treating the BMI adjustment as a sensitivity analysis.**

Response:

Thank you for this important comment. As suggested, we have revised the analysis to include BMI as a covariate in the primary model rather than treating it as part of a sensitivity analysis. The multivariable-adjusted HR was 1.25 (95% CI: 1.07–1.46, *p* = 0.005) per 1 SD decrease in the SOC score, which was consistent with the original findings.

**P11, L164–165:
Regarding the differences in participant characteristics based on the presence or absence of missing data, would it be possible to perform a statistical analysis to determine if significant differences exist between the two groups? Furthermore, please provide the SOC scores for these groups. Since the results for "S1" have been presented, they should also be addressed in the Discussion.**

Response:

We are grateful for your valuable suggestions regarding our manuscript. In response, we conducted statistical comparisons between participants with and without missing data, and the results are presented in S1 Table. Continuous variables were compared using the Wilcoxon rank-sum test, whereas categorical variables were compared using Pearson’s chi-square test. P values were presented for these comparisons. We have also provided SOC scores for both groups in the same table.

Furthermore, we have incorporated these findings into the Discussion section, addressing the potential implications of differences between the groups and their impact on the interpretation of our results.

“Fourth, there were missing data in the covariates, and participant characteristics differed between those with and without missing data (S1 Table). Specifically, those with missing data were older, less likely to have a current full-time job, and more likely to be widowed. Therefore, we cannot exclude the possibility that this may have affected the study results.” (lines 322-326, page 25 of the revised manuscript)

**P12:
What are the mean scores and distribution of SOC in this sample? Although SOC is the primary variable of interest, it is not a universally familiar metric. Please provide the mean and distribution of SOC scores, and present the differences in participant characteristics by SOC categories (e.g., divided into two groups by high/low or three groups based on standardized scores).**

Response:

We appreciate your insightful comments. We provided the mean and distribution of SOC scores, and present differences in participant characteristics by SOC categories (high, lower, lowest) in Table 1 as you suggested.

“The mean SOC score was 17.2 (standard deviation [SD], 4.2). The median SOC score for each SOC category is presented in Table 1.” (lines 196-198, pages 12-13 of the revised manuscript)

**P12:
It is highly plausible that SOC is significantly influenced by Socio-Economic Status (SES). In general, what is the relationship between SOC and SES? Was SES evaluated? If SOC is strongly influenced by SES, the lack of assessment or adjustment for SES would be a major limitation.**

Response:

Thank you for your helpful comments. We adjusted for employment status, but we could not measure socioeconomic status such as educational attainment and income. As you pointed out, SOC is influenced by socioeconomic status (SES), and this relationship may be bidirectional. We used social function of SF-36 and marital status for sensitivity analysis. The results of the sensitivity analysis which further adjusted these variables remained consistent.

“First, we additionally adjusted for marital status and the Social Functioning score of the 36-Item Short Form Health Survey (SF-36) to account for social factors in the relationship between SOC and the outcome [34]. These variables were not included in the primary analysis because social factors may act not only as confounders but also as potential mediators in the relationship between SOC and health outcomes, given the potentially bidirectional relationship between SOC and social factors [8].” (lines 158-163, pages 10-11 of the revised manuscript)

“First, we could not adjust for socioeconomic status such as educational attainment and income, which may have led to residual confounding. However, we adjusted for the most important covariates considered in previous studies (age, sex, comorbidities, employment status, smoking status, and alcohol consumption) [39,42]. Moreover, the sensitivity analyses additionally adjusting for marital status and the Social Functioning domain of the SF-36 showed consistent findings.” (lines 313-318, pages 24-25 of the revised manuscript)

**P14, 16 (Tables 2 and 3):
Please provide the number of participants (n) for each SOC category (High, Lower, Lowest).**

Response:

We are grateful for your valuable suggestion. In response, we have added the number of participants (n) in each SOC category (High, Lower, and Lowest) to Tables 2 and 3 in the manuscript.

**P19 (Discussion):
Please discuss the nature of this study's sample and its generalizability by comparing participant characteristics and SOC scores with those found in previous literature.**

Response:

We are grateful for your valuable suggestion. In response, we have expanded the Discussion to address the generalizability of our findings by comparing participant characteristics and SOC scores with those reported in previous literature.

Our cohort showed relatively higher SOC levels compared with prior literature, suggesting that participants may represent a healthier or more health-conscious population. We have discussed how this difference may affect the generalizability of our findings.

“Fifth, this study was conducted in only two rural towns in Japan, and it is unclear whether our results can be applied to other regions. The mean SOC score in this study was higher than that reported for the general population in Japan [35]. The study participants may have been more health-conscious than the general population, warranting caution when generalizing the results to other populations.” (lines 326-331, page 25 of the revised manuscript)

**P20, L259–260:
The manuscript states, "those with a high SOC are less likely to engage in high-risk behaviors, such as smoking and excessive alcohol consumption." Was this trend actually observed among the participants of this study?**

Response:

Thank you for this important comment. In our data, SOC was not associated with alcohol or smoking, possibly because we could not measure excessive smoking or excessive alcohol in detail. We referred to the prior literature regarding the relationship between SOC and health related behavior.

“Several underlying factors could influence this; for instance, those with a high SOC are previous studies have suggested that individuals with a high SOC may be less likely to engage in high-risk behaviors, such as smoking and excessive alcohol consumption [50, 51].” (lines 280-283, page 23 of the revised manuscript)

**P21, L277–285:
Please consider whether the discussion regarding the "modifiability" (improvement potential) of SOC would be better suited for the Introduction section.**

Response:

Thank you for this helpful suggestion. We agree that the modifiability of SOC is an important consideration. We have therefore incorporated this point into the Introduction section to better frame SOC as a potentially modifiable exposure.

“Although SOC has traditionally been conceptualized as relatively stable after early adulthood, accumulating evidence suggests that it may be modifiable through life experiences and programs at promoting self-care and self-management [9,10].” (lines 59-61, page 5 of the revised manuscript)

**P21, L289–291:
Since SES was mentioned, please elaborate on the relationship between SOC and SES in either the Background or Discussion. Additionally, regarding the statement, "However, we adjusted for the most important covariates considered in previous studies [36, 38]," please explicitly list which covariates are being referred to.**

Response:

We are grateful for your valuable suggestions regarding our manuscript. In response, we have expanded the Discussion to include the relationship between SOC and socioeconomic status (SES), based on prior literature.

“Childhood environments, such as lower socioeconomic status and limited social support, have been suggested to contribute to lower SOC in adolescence [59], and SOC has been considered relatively stable throughout adulthood.” (lines 300-303, page 24 of the revised manuscript)

We have also clarified which covariates have been adjusted for in previous studies by explicitly listing them in the manuscript.

“However, we adjusted for the most important covariates considered in previous studies (age, sex, comorbidities, employment status, smoking status, and alcohol consumption) [39,42].” (lines 315-317, page 24 of the revised manuscript)

**Figure 1:
Please consider including the number of participants lost to follow-up due to relocation in the flowchart.**

Response:

Thank you for your suggestion. We revised the flowchart in Figure 1 to include the number of participants lost to follow-up due to relocation. Those who were lost to follow-up due to relocation were 22, and we could complete the follow-up remaining 1,639 participants.

**Reviewer #2:**

**Reviewer #2: Thank you for the opportunity to review this manuscript. The topic is important, and the study addresses a clinically and socially relevant question regarding sense of coherence (SOC) and adverse health outcomes among older adults. The manuscript is generally well written. I offer the following comments in the hope that they will help improve the clarity, interpretation, and overall rigor of the study.**

Response:

We thank you for your thoughtful and constructive review of our manuscript. We appreciate your positive evaluation of the study and your helpful comments, which have greatly contributed to improving the clarity, interpretation, and overall rigor of the manuscript. We address each of your comments in detail below.

**Major comments

1. Potential residual confounding by social factors
Regarding the covariates described around lines 117 onward, I wonder whether the reported association may still be influenced by social confounding factors other than employment status, such as participation in social activities, community engagement, or other forms of social involvement. Could the authors clarify why these factors were not adjusted for in the analysis? If such variables are available, it would strengthen the manuscript to examine whether the observed association remains after adjustment for these potential confounders. If these data were not collected, or if adjustment for these variables is not appropriate because of concerns such as overadjustment or model distortion, I would encourage the authors to explicitly acknowledge this point in the limitations section.**

Response:

Thank you for your thoughtful comments. We agree that social factors could influence the relationship between SOC and outcomes. In our dataset, we could measure the social function of SF-36 and marital status, while we could not obtain educational status, income or social capital. We added the limitation that we could not adjust for community engagement, educational status and income explicitly. We also added a sensitivity analysis adjusting for the Social Functioning score of the 36-Item Short Form Survey and marital status, and the results were consistent.

“First, we additionally adjusted for marital status and the Social Functioning score of the 36-Item Short Form Health Survey (SF-36) to account for social factors in the relationship between SOC and the outcome [34]. These variables were not included in the primary analysis because social factors may act not only as confounders but also as potential mediators in the relationship between SOC and health outcomes, given the potentially bidirectional relationship between SOC and socioeconomic or social factors [8].” (lines 158-163, pages 10-11 of the revised manuscript)

“In the sensitivity analysis, the estimate was similar after additional adjustment for marital status and the Social Functioning score of the SF-36 (adjusted HR, 1.27 [95% CI, 1.08–1.49]; p = 0.004).” (lines 212-214, page 17 of the revised manuscript)

“First, we could not adjust for community engagement and socioeconomic status such as educational attainment and income, which may have led to residual confounding.” (lines 313-315, page 24 of the revised manuscript)

“Moreover, the sensitivity analyses additionally adjusting for marital status and the Social Functioning domain of the SF-36 showed consistent findings.” (lines 317-318, pages 24-25 of the revised manuscript)

**Minor comments

2.Categorization of employment status as a covariate
Employment status appears to have been handled as a binary factor, such as full-time job versus not. Given that many participants seem to be older adults well beyond their 70s, various forms of non-full-time work may also be common in this population. The authors are encouraged to explain the rationale for this categorization and to discuss the significance and limitations of adjusting for employment status in this manner. If feasible, it may also be informative to explore an analysis using a covariate specification that takes non-full-time work into account. However, if this is difficult within the scope of the current study, I do not think the authors need to force such an analysis.**

Response:

We are grateful to you for your valuable suggestions regarding our manuscript. As you mentioned, the employment status varied in this population, and we decided to take part-time job into account in the analysis. We categorized the “full-time” or “not” into “full-time, part-time, or other”.

“As potential confounders, we adjusted for age, sex, body mass index, employment status (having a current full-time job, having a part-time job or other), living alone or not, number of comorbidities, alcohol consumption (none, less than 20 g/day, 20 g/day or more), smoking status (current smoker or not), baseline mental health status, and baseline physical activity.” (lines 139-142, page 9 of the revised manuscript)

**3. Possibility that SOC may change over time
Regarding the description of sense of coherence (SOC) around lines 86 and 90 onward, since SOC appears to have been measured only at baseline, it may be helpful for the authors to discuss the possibility that SOC could change over time, particularly in older adults. I understand that formally addressing this issue may require substantial changes to the analytical framework. Therefore, if additional analyses are beyond the scope of the current study, I believe it would still be important to mention this point as a limitation.**

Response:

We appreciate this important comment. Because SOC was measured only once at baseline, a single measurement may not have fully captured long-term SOC status during follow-up. We have clarified this point in the limitations section.

“Finally, SOC was measured only once at baseline, and changes in SOC during follow-up could not be assessed.” (lines 331-332, page 25 of the revised manuscript)

**4. Description of the study setting
The manuscript includes participants from Minami-Aizu Town and Tadami Town in Fukushima Prefecture. Providing a brief description of these municipalities would help readers better understand the social and demographic background of the study population. For example, a short explanation of whether these are rural, aging, or geographically remote communities would improve the contextual interpretation of the results.**

Response:

We are grateful for your valuable suggestion. We have added a brief description of the study setting, including characteristics of Minami-Aizu Town and Tadami Town in Fukushima Prefecture, to provide context on the social and demographic background of the study population. These additions clarify that the study was conducted in relatively rural and aging communities, thereby improving the interpretability and generalizability of the findings.

“Tadami Town and Minami-Aizu Town are rural, mountainous regions, and the majority of participants were aged 70 years or older.” (lines 79-81, page 6 of the revised manuscript)

“Fifth, this study was conducted in only two rural towns in Japan, and it is unclear whether our results can be applied to other regions.” (lines 326-328, page 25 of the revised manuscript)

**I hope these comments are helpful to the authors in revising the manuscript.**

Response:

We appreciate all the comments, which in our opinion have greatly helped us improve the paper.
