# Supplementary material for: Association between sense of coherence and loss of independence or death in older adults: Locomotive Syndrome and Health Outcomes in Aizu Cohort Study
Source: PLoS One. 2026 Aug 31;21(8):e0355726. doi: 10.1371/journal.pone.0355726 (PMC13528987; doi:10.1371/journal.pone.0355726)
Supplement: Supplementary file 6 [file pone.0355726.s004.docx]

July 15, 2026

**Dear editors and reviewers**,

Thank you for your constructive comments and important suggestions. We appreciate the opportunity to resubmit our manuscript entitled, “Association between sense of coherence and loss of independence or death in older adults: Locomotive Syndrome and Health Outcomes in Aizu Cohort Study,” for publication in *PLOS One* as an original article. We have revised our paper in accordance with all the comments and helpful suggestions made by the editors and reviewers.

In accordance with your instructions, we have prepared the following files:

1) Point-by-point responses

2) Revised manuscript with track change to show the edits

3) Revised clean copy of our manuscript, figures, and supplementary materials

We feel that the revision has improved our manuscript and hope that it is now suitable for publication.

Kind regards,

**Yosuke Yamamoto, MD, PhD**

Department of Healthcare Epidemiology, Graduate School of Medicine, Kyoto University

54 Kawahara-cho, Syogoin, Sakyo-ku, Kyoto 606-8501, Japan

E-mail: [yamamoto.yosuke.5n@kyoto-u.ac.jp](mailto:yamamoto.yosuke.5n@kyoto-u.ac.jp)

**Reviewer #1: Major Comments
Marital status and the Social Functioning domain of the SF-36 are used as proxy indicators for socio-economic status (SES). However, neither of these necessarily reflects an individual's actual financial situation. A major limitation remains in that SES (such as economic status and educational background) has not been adequately accounted for.**

Response:

Thank you for pointing this out. We agree that marital status and the Social Functioning domain of the SF-36 do not directly measure socioeconomic status such as educational status or financial status. Direct measures of socioeconomic status, such as household income, educational attainment and assets, were not available in this cohort. Therefore, residual confounding by socioeconomic status cannot be excluded. We have revised the Discussion to explicitly acknowledge this limitation and have avoided interpreting marital status and Social Functioning as sufficient proxies for socioeconomic status.

“First, although we adjusted for key covariates identified in previous studies, including age, sex, comorbidities, employment status, smoking status, and alcohol consumption [39, 42], and obtained consistent findings in sensitivity analyses additionally adjusting for marital status and the Social Functioning domain of the SF-36, we could not directly measure socioeconomic status such as educational attainment and financial circumstances. Therefore, residual confounding by these unmeasured factors cannot be excluded.” (lines 312–318, pages 24–25 of the revised manuscript)

**Given that this study focuses on SOC, it is necessary to provide evidence regarding how robust this indicator is against the passage of time and fluctuations in the participants' emotional states.**

Response:

Thank you for this important comment. In a three-wave Japanese panel study, the SOC3-UTHS demonstrated longitudinal measurement invariance, with stability coefficients of 0.69 and 0.70 between consecutive annual assessments^1^. Previous longitudinal research using the SOC-13 has similarly indicated that SOC is relatively stable over time, although it may change following major life events^2^. In the present study, SOC was measured only at baseline; therefore, we could not directly assess within-person changes during follow-up. We have clarified the remaining limitation in the revised manuscript.

“Although SOC is considered a relatively stable orientation and we adjusted for baseline mental health using the MHI-5, the possible influence of emotional status due to major life events cannot be excluded [60].” (lines 331–334, page 25 of the revised manuscript)

Reference:

1. Togari Y. Examination of the reliability and validity of the University of Tokyo Health Sociology version of the SOC3 scale (SOC3-UTHS) using three-wave data from the Japanese Life Course Panel Survey [in Japanese]. Available from: [https://csrda.iss.u-tokyo.ac.jp/panel/dp/PanelDP_045Togari.pdf](https://csrda.iss.u-tokyo.ac.jp/panel/dp/PanelDP_045Togari.pdf?utm_source=chatgpt.com))
2. Lövheim H, Graneheim UH, Jonsén E, Strandberg G, Lundman B. Changes in sense of coherence in old age - a 5-year follow-up of the Umeå 85+ study. Scand J Caring Sci. 2013 Mar;27(1):13-9.

**Minor Comments
Regarding Table 1, I recommend performing a statistical analysis to examine the differences among the three groups.**

Response:

Thank you for this suggestion. We performed statistical comparisons of baseline characteristics across the three SOC groups and added the corresponding P values to Table 1. Continuous variables are compared using the Kruskal-Wallis test, and categorical variables are compared using Pearson’s chi-square test.

**The text states, "We collected the LTCI certification status, consisting of care levels and updated dates each year." Please describe the exact data collection method for the LOI (i.e., from where and how it was obtained).**

Response:

We have clarified the data collection method for the long-term care insurance outcome. Information on LTCI certification status, including certified care level and the dates of certification or subsequent updates, was obtained annually from administrative records maintained by the participating local governments.

**Reviewer #2: I appreciate the authors' careful and thorough revisions in response to the reviewers' comments. All the points I raised in the previous round have been adequately addressed, and the revised manuscript reads more clearly than before. I do not have substantial further concerns at this stage.**

Response:

Thank you for your positive assessment of our revisions. We appreciate your careful review and are pleased that our responses have adequately addressed your concerns and improved the clarity of the manuscript.
