# Supplementary material for: Association between sense of coherence and loss of independence or death in older adults: Locomotive Syndrome and Health Outcomes in Aizu Cohort Study
Source: PLoS One. 2026 Aug 31;21(8):e0355726. doi: 10.1371/journal.pone.0355726 (PMC13528987; doi:10.1371/journal.pone.0355726)
Supplement: Peer review history

# Peer review: Decision Letter 0

**DOI:** 10.1371/journal.pone.0355726.r001

**Authors:** Yujiro Kuroda

9 Apr 2026

Dear Dr. Yamamoto,

Thank you for submitting your manuscript to PLOS ONE. After careful consideration, we feel that it has merit but does not fully meet PLOS ONE’s publication criteria as it currently stands. Therefore, we invite you to submit a revised version of the manuscript that addresses the points raised during the review process.

Please submit your revised manuscript by May 24 2026 11:59PM. If you will need more time than this to complete your revisions, please reply to this message or contact the journal office at plosone@plos.org. When you're ready to submit your revision, log on to https://www.editorialmanager.com/pone/ and select the 'Submissions Needing Revision' folder to locate your manuscript file.

- A letter that responds to each point raised by the academic editor and reviewer(s). You should upload this letter as a separate file labeled 'Response to Reviewers'.
- A marked-up copy of your manuscript that highlights changes made to the original version. You should upload this as a separate file labeled 'Revised Manuscript with Track Changes'.
- An unmarked version of your revised paper without tracked changes. You should upload this as a separate file labeled 'Manuscript'.

If you would like to make changes to your financial disclosure, please include your updated statement in your cover letter. Guidelines for resubmitting your figure files are available below the reviewer comments at the end of this letter.

If applicable, we recommend that you deposit your laboratory protocols in protocols.io to enhance the reproducibility of your results. Protocols.io assigns your protocol its own identifier (DOI) so that it can be cited independently in the future. For instructions see: https://journals.plos.org/plosone/s/submission-guidelines#loc-laboratory-protocols. Additionally, PLOS ONE offers an option for publishing peer-reviewed Lab Protocol articles, which describe protocols hosted on protocols.io. Read more information on sharing protocols at https://plos.org/protocols?utm_medium=editorial-email&utm_source=authorletters&utm_campaign=protocols.

As the corresponding author, your ORCID iD is verified in the submission system and will appear in the published article. PLOS supports the use of ORCID, and we encourage all coauthors to register for an ORCID iD and use it as well. Please encourage your coauthors to verify their ORCID iD within the submission system before final acceptance, as unverified ORCID iDs will not appear in the published article. *Only*  the individual author can complete the verification step; PLOS staff *cannot*  verify ORCID iDs on behalf of authors.

We look forward to receiving your revised manuscript.

Kind regards,

Yujiro Kuroda

Academic Editor

PLOS One

Journal Requirements:

When submitting your revision, we need you to address these additional requirements.

1. Please ensure that your manuscript meets PLOS ONE's style requirements, including those for file naming. The PLOS ONE style templates can be found at

https://journals.plos.org/plosone/s/file?id=wjVg/PLOSOne_formatting_sample_main_body.pdf and

https://journals.plos.org/plosone/s/file?id=ba62/PLOSOne_formatting_sample_title_authors_affiliations.pdf.

2. We note that you have indicated that there are restrictions to data sharing for this study. For studies involving human research participant data or other sensitive data, we encourage authors to share de-identified or anonymized data. However, when data cannot be publicly shared for ethical reasons, we allow authors to make their data sets available upon request. For information on unacceptable data access restrictions, please see http://journals.plos.org/plosone/s/data-availability#loc-unacceptable-data-access-restrictions.

Before we proceed with your manuscript, please address the following prompts:

a) If there are ethical or legal restrictions on sharing a de-identified data set, please explain them in detail (e.g., data contain potentially identifying or sensitive patient information, data are owned by a third-party organization, etc.) and who has imposed them (e.g., a Research Ethics Committee or Institutional Review Board, etc.). Please also provide contact information for a data access committee, ethics committee, or other institutional body to which data requests may be sent.

b) If there are no restrictions, please upload the minimal anonymized data set necessary to replicate your study findings to a stable, public repository and provide us with the relevant URLs, DOIs, or accession numbers. Please see http://www.bmj.com/content/340/bmj.c181.long for guidelines on how to de-identify and prepare clinical data for publication. For a list of recommended repositories, please see https://journals.plos.org/plosone/s/recommended-repositories. You also have the option of uploading the data as Supporting Information files, but we would recommend depositing data directly to a data repository if possible.

Please update your Data Availability statement in the submission form accordingly.

3. If the reviewer comments include a recommendation to cite specific previously published works, please review and evaluate these publications to determine whether they are relevant and should be cited. There is no requirement to cite these works unless the editor has indicated otherwise.

Additional Editor Comments (if provided):

Dear Authors,

Thank you for submitting your manuscript examining the association between sense of coherence (SOC) and loss of independence or death among older adults. The study addresses an important research question and is supported by a well-defined cohort and longitudinal design. The reviewers found the topic to be relevant and the analysis generally appropriate.

However, after careful evaluation of the manuscript and reviewer comments, several issues require substantial clarification and improvement before the manuscript can be considered further. These primarily concern the reporting of key variables, handling of confounding (particularly social factors), and interpretability of the findings.

A major revision is therefore required.

1. Reporting of Exposure (SOC)

The reporting of the primary exposure is currently insufficient.

Please provide descriptive statistics for SOC, including:

- Mean and distribution

- Categorized presentation (e.g., tertiles or predefined groups)

- Participant characteristics by SOC category

Please clarify the measurement of SOC (SOC3-UTHS), including:

- Brief description of items (full wording optional if previously validated and cited)

- Evidence on reliability and validity (with quantitative indicators if available)

2. Confounding and Interpretation (Critical Issue)

The interpretation of the association between SOC and outcomes requires further strengthening.

The potential for residual confounding by socioeconomic and social factors needs more explicit treatment:

- Clarify whether SES variables were available

- If unavailable, discuss this more explicitly as a limitation

- Expand discussion on the relationship between SOC and SES based on prior literature

Similarly, please address the potential role of social participation or community engagement:

- If data are available, consider additional adjustment

- If not, acknowledge this as a limitation

3. Missing Data and Sample Selection

- Please report statistical comparisons between participants included and excluded due to missing data.

- Provide SOC values for these groups if possible.

- Ensure that findings from supplementary analyses (e.g., S1 Table) are incorporated into the Discussion where relevant.

4. Study Description and Transparency

Provide key cohort information within the manuscript (even if previously published), including:

- Recruitment process

- Follow-up duration

- Key inclusion/exclusion criteria

Expand the description of the study setting (Minami-Aizu and Tadami) to aid interpretation of generalizability.

In Figure 1, include the number of participants censored due to relocation.

5. Clarification of Analytical Choices

Provide clearer justification for:

- The treatment of BMI as a sensitivity analysis

- The categorization of employment status

Clarify which covariates are referred to as “important covariates in previous studies.”

6. Interpretation and Discussion

Ensure that statements regarding behavioral mechanisms (e.g., smoking, alcohol use) are:

- Supported by your data, or

- Clearly referenced as prior evidence

Discuss the possibility that SOC may change over time, given that it was measured only at baseline.

Strengthen the discussion of generalizability by comparing your findings and SOC levels with previous studies.

Summary

The manuscript has clear potential, but key aspects of reporting and interpretation require improvement. In particular, clarification of SOC measurement, handling of social confounding, and presentation of descriptive results will substantially strengthen the paper.

We encourage a careful and structured revision with a point-by-point response.

[Note: HTML markup is below. Please do not edit.]

Reviewers' comments:

Reviewer's Responses to Questions

**Comments to the Author**

1. Is the manuscript technically sound, and do the data support the conclusions?

Reviewer #1: Partly

Reviewer #2: Yes

**********

2. Has the statistical analysis been performed appropriately and rigorously?

Reviewer #1: Yes

Reviewer #2: Yes

**********

3. Have the authors made all data underlying the findings in their manuscript fully available?

The PLOS Data policy

Reviewer #1: Yes

Reviewer #2: Yes

**********

4. Is the manuscript presented in an intelligible fashion and written in standard English?

Reviewer #1: Yes

Reviewer #2: Yes

**********

Reviewer #1: Reviewer Comments

This is a valuable study focusing on the Sense of Coherence (SOC)—a pivotal capacity for making healthy behavioral choices—and demonstrating its association with critical outcomes such as the need for long-term care and mortality. The study's significance is further bolstered by the use of a large-scale, long-term longitudinal dataset. However, certain essential information and results are currently missing. A final decision will require the following points to be addressed through clarification or supplementation.

Specific Comments

P6 (Methods: Design and setting / Participants):

While previous studies are cited, please provide the minimum essential protocol information for the cohort study within this manuscript (e.g., age range of participants, total sample size, follow-up period, and recruitment methods).

P7 (SOC3-UTHS):

Please include the specific items of the SOC3-UTHS in the text. Additionally, provide the reliability and validity scores for this scale.

(Note to the author: Separately, please clarify for the reviewer’s understanding—not necessarily for inclusion in the text—what criteria were used to establish the validity of the SOC3-UTHS, or its parent SOC scale. Specifically, what serves as the "gold standard" to confirm it truly measures SOC?)

P9, L137–138:

It is unclear why a separate analysis focusing solely on BMI is necessary. Could it not be included in the primary analysis from the outset? Please explain the rationale for treating the BMI adjustment as a sensitivity analysis.

P11, L164–165:

Regarding the differences in participant characteristics based on the presence or absence of missing data, would it be possible to perform a statistical analysis to determine if significant differences exist between the two groups? Furthermore, please provide the SOC scores for these groups. Since the results for "S1" have been presented, they should also be addressed in the Discussion.

P12:

What are the mean scores and distribution of SOC in this sample? Although SOC is the primary variable of interest, it is not a universally familiar metric. Please provide the mean and distribution of SOC scores, and present the differences in participant characteristics by SOC categories (e.g., divided into two groups by high/low or three groups based on standardized scores).

P12:

It is highly plausible that SOC is significantly influenced by Socio-Economic Status (SES). In general, what is the relationship between SOC and SES? Was SES evaluated? If SOC is strongly influenced by SES, the lack of assessment or adjustment for SES would be a major limitation.

P14, 16 (Tables 2 and 3):

Please provide the number of participants (n) for each SOC category (High, Lower, Lowest).

P19 (Discussion):

Please discuss the nature of this study's sample and its generalizability by comparing participant characteristics and SOC scores with those found in previous literature.

P20, L259–260:

The manuscript states, "those with a high SOC are less likely to engage in high-risk behaviors, such as smoking and excessive alcohol consumption." Was this trend actually observed among the participants of this study?

P21, L277–285:

Please consider whether the discussion regarding the "modifiability" (improvement potential) of SOC would be better suited for the Introduction section.

P21, L289–291:

Since SES was mentioned, please elaborate on the relationship between SOC and SES in either the Background or Discussion. Additionally, regarding the statement, "However, we adjusted for the most important covariates considered in previous studies [36, 38]," please explicitly list which covariates are being referred to.

Figure 1:

Please consider including the number of participants lost to follow-up due to relocation in the flowchart.

Reviewer #2: Thank you for the opportunity to review this manuscript. The topic is important, and the study addresses a clinically and socially relevant question regarding sense of coherence (SOC) and adverse health outcomes among older adults. The manuscript is generally well written.I offer the following comments in the hope that they will help improve the clarity, interpretation, and overall rigor of the study.

Major comments

1. Potential residual confounding by social factors

Regarding the covariates described around lines 117 onward, I wonder whether the reported association may still be influenced by social confounding factors other than employment status, such as participation in social activities, community engagement, or other forms of social involvement. Could the authors clarify why these factors were not adjusted for in the analysis? If such variables are available, it would strengthen the manuscript to examine whether the observed association remains after adjustment for these potential confounders. If these data were not collected, or if adjustment for these variables is not appropriate because of concerns such as overadjustment or model distortion, I would encourage the authors to explicitly acknowledge this point in the limitations section.

Minor comments

2.Categorization of employment status as a covariate

Employment status appears to have been handled as a binary factor, such as full-time job versus not. Given that many participants seem to be older adults well beyond their 70s, various forms of non-full-time work may also be common in this population. The authors are encouraged to explain the rationale for this categorization and to discuss the significance and limitations of adjusting for employment status in this manner. If feasible, it may also be informative to explore an analysis using a covariate specification that takes non-full-time work into account. However, if this is difficult within the scope of the current study, I do not think the authors need to force such an analysis.

3. Possibility that SOC may change over time

Regarding the description of sense of coherence (SOC) around lines 86 and 90 onward, since SOC appears to have been measured only at baseline, it may be helpful for the authors to discuss the possibility that SOC could change over time, particularly in older adults. I understand that formally addressing this issue may require substantial changes to the analytical framework. Therefore, if additional analyses are beyond the scope of the current study, I believe it would still be important to mention this point as a limitation.

4. Description of the study setting

The manuscript includes participants from Minami-Aizu Town and Tadami Town in Fukushima Prefecture. Providing a brief description of these municipalities would help readers better understand the social and demographic background of the study population. For example, a short explanation of whether these are rural, aging, or geographically remote communities would improve the contextual interpretation of the results.

I hope these comments are helpful to the authors in revising the manuscript.

**********

what does this mean?). If published, this will include your full peer review and any attached files.

If you choose “no”, your identity will remain anonymous but your review may still be made public.

**Do you want your identity to be public for this peer review?**  For information about this choice, including consent withdrawal, please see our Privacy Policy

Reviewer #1: No

Reviewer #2: No

**********

[NOTE: If reviewer comments were submitted as an attachment file, they will be attached to this email and accessible via the submission site. Please log into your account, locate the manuscript record, and check for the action link "View Attachments". If this link does not appear, there are no attachment files.]

To ensure your figures meet our technical requirements, please review our figure guidelines: https://journals.plos.org/plosone/s/figures

You may also use PLOS’s free figure tool, NAAS, to help you prepare publication quality figures: https://journals.plos.org/plosone/s/figures#loc-tools-for-figure-preparation.

NAAS will assess whether your figures meet our technical requirements by comparing each figure against our figure specifications.

# Author comment: Author response to Decision Letter 1

**DOI:** 10.1371/journal.pone.0355726.r002

8 Jun 2026

May 24, 2026

Dear editors and reviewers,

Thank you for your constructive comments and important suggestions. We appreciate the opportunity to resubmit our manuscript entitled, “Association between sense of coherence and loss of independence or death in older adults: Locomotive Syndrome and Health Outcomes in Aizu Cohort Study,” for publication in PLOS One as an original article. We have revised our paper in accordance with all the comments and helpful suggestions made by the editors and reviewers.

In accordance with your instructions, we have prepared the following files:

1) Point-by-point responses

2) Revised manuscript with track change to show the edits

3) Revised clean copy of our manuscript, figures, and supplementary materials

We feel that the revision has improved our manuscript and hope that it is now suitable for publication.

Kind regards,

Yosuke Yamamoto, MD, PhD

Department of Healthcare Epidemiology, Graduate School of Medicine, Kyoto University

54 Kawahara-cho, Syogoin, Sakyo-ku, Kyoto 606-8501, Japan

E-mail: yamamoto.yosuke.5n@kyoto-u.ac.jp

POINT-BY-POINT RESPONSES TO EDITORS’ AND REVIEWERS’ COMMENTS:

Editor:

Dear Authors,

Thank you for submitting your manuscript examining the association between sense of coherence (SOC) and loss of independence or death among older adults. The study addresses an important research question and is supported by a well-defined cohort and longitudinal design. The reviewers found the topic to be relevant and the analysis generally appropriate.

However, after careful evaluation of the manuscript and reviewer comments, several issues require substantial clarification and improvement before the manuscript can be considered further. These primarily concern the reporting of key variables, handling of confounding (particularly social factors), and interpretability of the findings.

A major revision is therefore required.

Response:

Thank you for your interest in our work and for your helpful comments. We have revised the manuscript and responded point-by-point.

1. Reporting of Exposure (SOC)

The reporting of the primary exposure is currently insufficient.

Please provide descriptive statistics for SOC, including:

- Mean and distribution

- Categorized presentation (e.g., tertiles or predefined groups)

- Participant characteristics by SOC category

Response:

Thank you for your comment regarding the reporting of SOC. We revised the manuscript to address each of these points regarding the presentation of SOC.

- Mean and distribution

We added descriptive statistics of SOC in the Results section, including the mean and standard deviation:

“The mean SOC score was 17.2 (standard deviation [SD], 4.2). The median SOC score for each SOC category is presented in Table 1.” (lines 196–198, pages 12-13 of the revised manuscript).

- Categorized presentation

SOC was categorized into three groups based on standardized scores for descriptive and comparative analyses, and the median and interquartile ranges across these SOC categories were added to Table 1.

- Participant characteristics by SOC category

We revised Table 1 to present participant characteristics stratified by SOC category.

Please clarify the measurement of SOC (SOC3-UTHS), including:

- Brief description of items (full wording optional if previously validated and cited)

- Evidence on reliability and validity (with quantitative indicators if available)

Response:

Thank you for your thoughtful comments regarding the measurement of SOC. We have revised the manuscript to address each of these points.

- Brief description of items

We clarified the measurement of SOC by adding a brief description of the SOC-3-UTHS items in the Methods section:

“The SOC-3-UTHS is a Japanese short version of the SOC scale, comprising three items that address manageability (“I am able to find solutions to the hardships and problems that occur every day”), meaningfulness (“I think it is worth facing and dealing with some of the hardships and problems of life”), and comprehensibility (“I am able to understand and predict the hardships and problems that occur every day”) [26].” (lines 109–113, pages 7–8 of the revised manuscript).

- Evidence on reliability and validity

We added information regarding the psychometric properties of the SOC-3-UTHS, including previously reported reliability and validity indices:

“The validity and reliability of SOC-3-UTHS have been shown in a previous study, reporting good internal consistency (Cronbach’s α = 0.84) and a moderate correlation with the SOC-13 (r = 0.51) [25].” (lines 115–117, page 8 of the revised manuscript)

2. Confounding and Interpretation (Critical Issue)

The interpretation of the association between SOC and outcomes requires further strengthening.

The potential for residual confounding by socioeconomic and social factors needs more explicit treatment:

- Clarify whether SES variables were available

- If unavailable, discuss this more explicitly as a limitation

Response:

Thank you for highlighting the important issue of residual confounding by socioeconomic and social factors. We clarified that data on educational attainment and income were not available in our dataset, and we explicitly acknowledged this as a limitation of the study. As proxies for socioeconomic and social factors, we used marital status and the Social Functioning domain of the 36-Item Short Form Health Survey (SF-36), and additionally adjusted for these variables in a sensitivity analysis. The estimate was similar after additional adjustment (adjusted HR, 1.27 [95% CI, 1.08–1.49]; p = 0.004).

“First, we additionally adjusted for marital status and the Social Functioning score of the 36-Item Short Form Health Survey (SF-36) to account for social factors in the relationship between SOC and the outcome [34].” (lines 158-160, page 10 of the revised manuscript)

“In the sensitivity analysis, the estimate was similar after additional adjustment for marital status and the Social Functioning score of the SF-36 (adjusted HR, 1.27 [95% CI, 1.08–1.49]; p = 0.004).” (lines 212-214, page 17 of the revised manuscript)

“First, we could not adjust for socioeconomic status such as educational attainment and income, which may have led to residual confounding. However, we adjusted for the most important covariates considered in previous studies (age, sex, comorbidities, employment status, smoking status, and alcohol consumption) [39,42]. Moreover, the sensitivity analyses additionally adjusting for marital status and the Social Functioning domain of the SF-36 showed consistent findings.” (lines 313-318, pages 24-25 of the revised manuscript)

- Expand discussion on the relationship between SOC and SES based on prior literature

Response:

Thank you for this important suggestion. We expanded our discussion regarding the relationship between SOC and socioeconomic status (SES) based on prior literature. Childhood environments, such as lower SES have been suggested to contribute to lower SOC in adolescence (Soc Psychiatry Psychiatr Epidemiol. 2009 Dec;44(12):1097-105.). On the other hand, higher SOC has been suggested to help gain social relationships (The Handbook of Salutogenesis [Internet]. 2nd ed. Cham (CH): Springer. 2022). Therefore, it was difficult to define SES as confounder or mediators. We explicitly discussed this point in the Method and Discussion section.

“First, we additionally adjusted for marital status and the Social Functioning score of the 36-Item Short Form Health Survey (SF-36) to account for social factors in the relationship between SOC and the outcome [34]. These variables were not included in the primary analysis because social factors may act not only as confounders but also as potential mediators in the relationship between SOC and health outcomes, given the potentially bidirectional relationship between SOC and social factors [8].” (lines 158-163, pages 10-11 of the revised manuscript)

“Childhood environments, such as lower socioeconomic status and limited social support, have been suggested to contribute to lower SOC in adolescence [59], and SOC has been considered relatively stable throughout adulthood. However, recent studies have suggested that SOC can be modifiable [43, 60].” (lines 300-304, page 24 of the revised manuscript)

Similarly, please address the potential role of social participation or community engagement:

- If data are available, consider additional adjustment

- If not, acknowledge this as a limitation

Response:

Thank you for this valuable suggestion. While we could not directly measure “social participation” or “community engagement,” we had data regarding the Social Functioning domain of SF-36 (Med Care. 1992;30:473–483.) We conducted an additional sensitivity analysis further adjusting for marital status and the Social Functioning domain of the SF-36, and the results remained consistent.

“First, we additionally adjusted for marital status and the Social Functioning score of the 36-Item Short Form Health Survey (SF-36) to account for social factors in the relationship between SOC and the outcome [34].” (lines 158-160, page 10 of the revised manuscript)

“In the sensitivity analysis, the estimate was similar after additional adjustment for marital status and the Social Functioning score of the SF-36 (adjusted HR, 1.27 [95% CI, 1.08–1.49]; p = 0.004).” (lines 212-214, page 17 of the revised manuscript)

We also added the limitation that community engagement was not measured in the revised manuscript.

“First, we could not adjust for community engagement and socioeconomic status such as educational attainment and income, which may have led to residual confounding.” (lines 313-315, page 24 of the revised manuscript)

3. Missing Data and Sample Selection

- Please report statistical comparisons between participants included and excluded due to missing data.

- Provide SOC values for these groups if possible.

Response:

Thank you for highlighting the importance of assessing potential bias due to missing data.

- Please report statistical comparisons between participants included and excluded due to missing data.

We added statistical comparisons between participants included in the analysis and those excluded due to missing data, which are presented in S1 Table.

- Provide SOC values for these groups if possible.

We have provided SOC values for both included and excluded participants in S1 Table.

- Ensure that findings from supplementary analyses (e.g., S1 Table) are incorporated into the Discussion where relevant.

We have incorporated the findings from S1 Table into the Discussion section. Participants with missing data were older, less likely to have a current full-time job, and more likely to be widowed. We have discussed how these differences may have influenced the observed associations.

“Fourth, there were missing data in the covariates, and participant characteristics differed between those with and without missing data (S1 Table). Specifically, those with missing data were older, less likely to have a current full-time job, and more likely to be widowed. Therefore, we cannot exclude the possibility that this may have affected the study results.” (lines 322-326, page 25 of the revised manuscript)”

4. Study Description and Transparency

Provide key cohort information within the manuscript (even if previously published), including:

- Recruitment process

- Follow-up duration

- Key inclusion/exclusion criteria

Response:

Thank you for your helpful suggestions to improve the clarity and completeness of the cohort description. We have provided the key cohort information.

- Recruitment process

We added details regarding participant recruitment process.

“The study recruited community-dwelling participants aged 40–80 years who were National Health Insurance beneficiaries and participated in annual health checkups conducted by their local governments between 2008 and 2010 [23].”(lines 82–85, page 6 of the revised manuscript)

- Follow-up duration

We clarified the prospective follow-up process and current study status.

“After the baseline examination, participants were followed prospectively, and the study is currently ongoing.” (lines 91–92, page 6 of the revised manuscript)

- Key inclusion/exclusion criteria

We added key information regarding the study population.

“Survey forms were distributed to individuals attending the health checkups, and those who agreed to participate in the study returned the forms (the response rate was 60% in the first year) [23]. Approximately 5,800 individuals were recruited between 2008 and 2010 [24]. The baseline survey consisted of self-administered questionnaires, health checkups including blood and urine tests, musculoskeletal examinations, assessments of motor dysfunction, and radiographic assessments [23].” (lines 85–91, page 6 of the revised manuscript)

Expand the description of the study setting (Minami-Aizu and Tadami) to aid interpretation of generalizability.

Response:

We have expanded the description of the study setting, including characteristics of Minami-Aizu and Tadami, to provide better context for interpreting the generalizability of our findings.

“Tadami Town and Minami-Aizu Town are rural, mountainous regions, and the majority of participants were aged 70 years or older.” (lines 79-81, page 6 of the revised manuscript)

In Figure 1, include the number of participants censored due to relocation.

Response:

Thank you for pointing this out. We have revised Figure 1 to include the number of participants censored due to relocation. The number of participants lost to follow-up was 22, and the remaining 1,639 participants completed the follow-up.

5. Clarification of Analytical Choices

Provide clearer justification for:

- The treatment of BMI as a sensitivity analysis

- The categorization of employment status

Response:

Thank you for these important comments regarding our analytical choices. Following the reviewers’ suggestions, we revised the primary model by including BMI and modifying the categorization of employment status. The results remained statistically significant.

- The treatment of BMI as a sensitivity analysis

In the original analysis, BMI was included as part of a sensitivity analysis due to the controversy in relationship with SOC. However, following the reviewers’ suggestions, we reconsidered this approach and have now included BMI as a covariate in the primary analysis. The multivariable-adjusted HR was 1.25 (95% CI: 1.07–1.46, p = 0.005) per 1 SD decrease in the SOC score, which was consistent with the original findings.

- The categorization of employment status

Employment status was initially dichotomized as “full-time” versus “not full-time” to reflect the relatively high proportion of participants engaged in full-time work. However, to better capture heterogeneity in employment, we have revised the categorization to “full-time,” “part-time,” and “other.” The multivariable-adjusted HR was 1.25 (95% CI: 1.07–1.46, p = 0.005) per 1 SD decrease in the SOC score, which was consistent with the original findings.

“As potential confounders, we adjusted for age, sex, body mass index, employment status (having a current full-time job, having a part-time job or other), living alone or not, number of comorbidities, alcohol consumption (none, less than 20 g/day, 20 g/day or more), smoking status (current smoker or not), baseline mental health status, and baseline physical activity.” (lines 139-142, page 9 of the revised manuscript)

“Furthermore, multivariable analysis showed a similar association between a lower SOC and a higher risk of LOI or death. The multivariable-adjusted HR was 1.25 (95% CI: 1.07–1.46, p = 0.005) per 1 SD decrease in the SOC score. (lines 206-208, pages 14-15 of the revised manuscript)

Clarify which covariates are referred to as “important covariates in previous studies.”

Response:

Thank you for raising this point. We have clarified which covariates were considered important based on prior literature by explicitly listing them in the Discussion section.

“However, we adjusted for the most important covariates considered in previous studies (age, sex, comorbiditie

Attachment Submitted filename: Point_by_point_responses.docx

# Peer review: Decision Letter 1

**DOI:** 10.1371/journal.pone.0355726.r003

**Authors:** Yujiro Kuroda

10 Jul 2026

Dear Dr. Yamamoto,

Thank you for submitting your manuscript to PLOS ONE. After careful consideration, we feel that it has merit but does not fully meet PLOS ONE’s publication criteria as it currently stands. Therefore, we invite you to submit a revised version of the manuscript that addresses the points raised during the review process.

Please submit your revised manuscript by Aug 24 2026 11:59PM. If you will need more time than this to complete your revisions, please reply to this message or contact the journal office at plosone@plos.org. When you're ready to submit your revision, log on to https://www.editorialmanager.com/pone/ and select the 'Submissions Needing Revision' folder to locate your manuscript file.

- A letter that responds to each point raised by the academic editor and reviewer(s). You should upload this letter as a separate file labeled 'Response to Reviewers'.
- A marked-up copy of your manuscript that highlights changes made to the original version. You should upload this as a separate file labeled 'Revised Manuscript with Track Changes'.
- An unmarked version of your revised paper without tracked changes. You should upload this as a separate file labeled 'Manuscript'.

If you would like to make changes to your financial disclosure, please include your updated statement in your cover letter. Guidelines for resubmitting your figure files are available below the reviewer comments at the end of this letter.

If applicable, we recommend that you deposit your laboratory protocols in protocols.io to enhance the reproducibility of your results. Protocols.io assigns your protocol its own identifier (DOI) so that it can be cited independently in the future. For instructions see: https://journals.plos.org/plosone/s/submission-guidelines#loc-laboratory-protocols. Additionally, PLOS ONE offers an option for publishing peer-reviewed Lab Protocol articles, which describe protocols hosted on protocols.io. Read more information on sharing protocols at https://plos.org/protocols?utm_medium=editorial-email&utm_source=authorletters&utm_campaign=protocols.

As the corresponding author, your ORCID iD is verified in the submission system and will appear in the published article. PLOS supports the use of ORCID, and we encourage all coauthors to register for an ORCID iD and use it as well. Please encourage your coauthors to verify their ORCID iD within the submission system before final acceptance, as unverified ORCID iDs will not appear in the published article. *Only*  the individual author can complete the verification step; PLOS staff *cannot*  verify ORCID iDs on behalf of authors.

We look forward to receiving your revised manuscript.

Kind regards,

Yujiro Kuroda

Academic Editor

PLOS One

Journal Requirements:

1. If the reviewer comments include a recommendation to cite specific previously published works, please review and evaluate these publications to determine whether they are relevant and should be cited. There is no requirement to cite these works unless the editor has indicated otherwise.

2. Please review your reference list to ensure that it is complete and correct. If you have cited papers that have been retracted, please include the rationale for doing so in the manuscript text, or remove these references and replace them with relevant current references. Any changes to the reference list should be mentioned in the rebuttal letter that accompanies your revised manuscript. If you need to cite a retracted article, indicate the article’s retracted status in the References list and also include a citation and full reference for the retraction notice.

**Additional Editor Comments:**

Dear Authors,

Thank you for submitting the revised version of your manuscript. The revised manuscript has now been evaluated by the reviewers. While one reviewer recommended acceptance, another reviewer raised remaining concerns that should be addressed before a final decision can be made.

I therefore invite you to submit a further revised manuscript. In your revision, please respond carefully to the remaining reviewer comments and revise the manuscript as appropriate. If you believe that a requested change is not necessary or not feasible, please provide a clear explanation in your point-by-point response.

Please submit a detailed response letter together with a revised manuscript in which the changes are clearly indicated.

I look forward to receiving your revised manuscript.

[Note: HTML markup is below. Please do not edit.]

Reviewers' comments:

Reviewer's Responses to Questions

**Comments to the Author**

Reviewer #1: All comments have been addressed

Reviewer #2: (No Response)

**********

2. Is the manuscript technically sound, and do the data support the conclusions?

Reviewer #1: Yes

Reviewer #2: (No Response)

**********

3. Has the statistical analysis been performed appropriately and rigorously?

Reviewer #1: Yes

Reviewer #2: (No Response)

**********

4. Have the authors made all data underlying the findings in their manuscript fully available?

The PLOS Data policy

Reviewer #1: No

Reviewer #2: (No Response)

**********

5. Is the manuscript presented in an intelligible fashion and written in standard English?

Reviewer #1: Yes

Reviewer #2: (No Response)

**********

Reviewer #1: Major Comments

Marital status and the Social Functioning domain of the SF-36 are used as proxy indicators for socio-economic status (SES). However, neither of these necessarily reflects an individual's actual financial situation. A major limitation remains in that SES (such as economic status and educational background) has not been adequately accounted for.

Given that this study focuses on SOC, it is necessary to provide evidence regarding how robust this indicator is against the passage of time and fluctuations in the participants' emotional states.

Minor Comments

Regarding Table 1, I recommend performing a statistical analysis to examine the differences among the three groups.

The text states, "We collected the LTCI certification status, consisting of care levels and updated dates each year." Please describe the exact data collection method for the LOI (i.e., from where and how it was obtained).

Reviewer #2: I appreciate the authors' careful and thorough revisions in response to the reviewers' comments. All the points I raised in the previous round have been adequately addressed, and the revised manuscript reads more clearly than before. I do not have substantial further concerns at this stage.

**********

what does this mean?). If published, this will include your full peer review and any attached files.

If you choose “no”, your identity will remain anonymous but your review may still be made public.

**Do you want your identity to be public for this peer review?**  For information about this choice, including consent withdrawal, please see our Privacy Policy

Reviewer #1: No

Reviewer #2: No

**********

[NOTE: If reviewer comments were submitted as an attachment file, they will be attached to this email and accessible via the submission site. Please log into your account, locate the manuscript record, and check for the action link "View Attachments". If this link does not appear, there are no attachment files.]

To ensure your figures meet our technical requirements, please review our figure guidelines: https://journals.plos.org/plosone/s/figures

You may also use PLOS’s free figure tool, NAAS, to help you prepare publication quality figures: https://journals.plos.org/plosone/s/figures#loc-tools-for-figure-preparation.

NAAS will assess whether your figures meet our technical requirements by comparing each figure against our figure specifications.

# Author comment: Author response to Decision Letter 2

**DOI:** 10.1371/journal.pone.0355726.r004

15 Jul 2026

Reviewer #1: Major Comments

Marital status and the Social Functioning domain of the SF-36 are used as proxy indicators for socio-economic status (SES). However, neither of these necessarily reflects an individual's actual financial situation. A major limitation remains in that SES (such as economic status and educational background) has not been adequately accounted for.

Response:

Thank you for pointing this out. We agree that marital status and the Social Functioning domain of the SF-36 do not directly measure socioeconomic status such as educational status or financial status. Direct measures of socioeconomic status, such as household income, educational attainment and assets, were not available in this cohort. Therefore, residual confounding by socioeconomic status cannot be excluded. We have revised the Discussion to explicitly acknowledge this limitation and have avoided interpreting marital status and Social Functioning as sufficient proxies for socioeconomic status.

“First, although we adjusted for key covariates identified in previous studies, including age, sex, comorbidities, employment status, smoking status, and alcohol consumption [39, 42], and obtained consistent findings in sensitivity analyses additionally adjusting for marital status and the Social Functioning domain of the SF-36, we could not directly measure socioeconomic status such as educational attainment and financial circumstances. Therefore, residual confounding by these unmeasured factors cannot be excluded.” (lines 312–318, pages 24–25 of the revised manuscript)

Given that this study focuses on SOC, it is necessary to provide evidence regarding how robust this indicator is against the passage of time and fluctuations in the participants' emotional states.

Response:

Thank you for this important comment. In a three-wave Japanese panel study, the SOC3-UTHS demonstrated longitudinal measurement invariance, with stability coefficients of 0.69 and 0.70 between consecutive annual assessments1. Previous longitudinal research using the SOC-13 has similarly indicated that SOC is relatively stable over time, although it may change following major life events2. In the present study, SOC was measured only at baseline; therefore, we could not directly assess within-person changes during follow-up. We have clarified the remaining limitation in the revised manuscript.

“Although SOC is considered a relatively stable orientation and we adjusted for baseline mental health using the MHI-5, the possible influence of emotional status due to major life events cannot be excluded [60].” (lines 331–334, page 25 of the revised manuscript)

Reference:

1. Togari Y. Examination of the reliability and validity of the University of Tokyo Health Sociology version of the SOC3 scale (SOC3-UTHS) using three-wave data from the Japanese Life Course Panel Survey [in Japanese]. Available from: https://csrda.iss.u-tokyo.ac.jp/panel/dp/PanelDP_045Togari.pdf)

2. Lövheim H, Graneheim UH, Jonsén E, Strandberg G, Lundman B. Changes in sense of coherence in old age - a 5-year follow-up of the Umeå 85+ study. Scand J Caring Sci. 2013 Mar;27(1):13-9.

Minor Comments

Regarding Table 1, I recommend performing a statistical analysis to examine the differences among the three groups.

Response:

Thank you for this suggestion. We performed statistical comparisons of baseline characteristics across the three SOC groups and added the corresponding P values to Table 1. Continuous variables are compared using the Kruskal-Wallis test, and categorical variables are compared using Pearson’s chi-square test.

The text states, "We collected the LTCI certification status, consisting of care levels and updated dates each year." Please describe the exact data collection method for the LOI (i.e., from where and how it was obtained).

Response:

We have clarified the data collection method for the long-term care insurance outcome. Information on LTCI certification status, including certified care level and the dates of certification or subsequent updates, was obtained annually from administrative records maintained by the participating local governments.

Reviewer #2: I appreciate the authors' careful and thorough revisions in response to the reviewers' comments. All the points I raised in the previous round have been adequately addressed, and the revised manuscript reads more clearly than before. I do not have substantial further concerns at this stage.

Response:

Thank you for your positive assessment of our revisions. We appreciate your careful review and are pleased that our responses have adequately addressed your concerns and improved the clarity of the manuscript.

Attachment Submitted filename: point_by_point_responses_0715.docx

# Editor report: Decision Letter 2

**DOI:** 10.1371/journal.pone.0355726.r005

**Authors:** Yujiro Kuroda

16 Jul 2026

Dear Dr. Yamamoto,

Thank you for submitting your manuscript to PLOS ONE. After careful consideration, we feel that it has merit but does not fully meet PLOS ONE’s publication criteria as it currently stands. Therefore, we invite you to submit a revised version of the manuscript that addresses the points raised during the review process.

Please submit your revised manuscript by Aug 30 2026 11:59PM. If you will need more time than this to complete your revisions, please reply to this message or contact the journal office at plosone@plos.org. When you're ready to submit your revision, log on to https://www.editorialmanager.com/pone/ and select the 'Submissions Needing Revision' folder to locate your manuscript file.

- A letter that responds to each point raised by the academic editor and reviewer(s). You should upload this letter as a separate file labeled 'Response to Reviewers'.
- A marked-up copy of your manuscript that highlights changes made to the original version. You should upload this as a separate file labeled 'Revised Manuscript with Track Changes'.
- An unmarked version of your revised paper without tracked changes. You should upload this as a separate file labeled 'Manuscript'.

If you would like to make changes to your financial disclosure, please include your updated statement in your cover letter. Guidelines for resubmitting your figure files are available below the reviewer comments at the end of this letter.

If applicable, we recommend that you deposit your laboratory protocols in protocols.io to enhance the reproducibility of your results. Protocols.io assigns your protocol its own identifier (DOI) so that it can be cited independently in the future. For instructions see: https://journals.plos.org/plosone/s/submission-guidelines#loc-laboratory-protocols. Additionally, PLOS ONE offers an option for publishing peer-reviewed Lab Protocol articles, which describe protocols hosted on protocols.io. Read more information on sharing protocols at https://plos.org/protocols?utm_medium=editorial-email&utm_source=authorletters&utm_campaign=protocols.

As the corresponding author, your ORCID iD is verified in the submission system and will appear in the published article. PLOS supports the use of ORCID, and we encourage all coauthors to register for an ORCID iD and use it as well. Please encourage your coauthors to verify their ORCID iD within the submission system before final acceptance, as unverified ORCID iDs will not appear in the published article. *Only*  the individual author can complete the verification step; PLOS staff *cannot*  verify ORCID iDs on behalf of authors.

We look forward to receiving your revised manuscript.

Kind regards,

Yujiro Kuroda

Academic Editor

PLOS One

Journal Requirements:

If the reviewer comments include a recommendation to cite specific previously published works, please review and evaluate these publications to determine whether they are relevant and should be cited. There is no requirement to cite these works unless the editor has indicated otherwise.

Please review your reference list to ensure that it is complete and correct. If you have cited papers that have been retracted, please include the rationale for doing so in the manuscript text, or remove these references and replace them with relevant current references. Any changes to the reference list should be mentioned in the rebuttal letter that accompanies your revised manuscript. If you need to cite a retracted article, indicate the article’s retracted status in the References list and also include a citation and full reference for the retraction notice.

Additional Editor Comments:

Dear Authors,

Thank you for submitting the revised manuscript and the detailed point-by-point response.

The reviewers’ evaluations differed in the previous round, with one reviewer recommending acceptance and the other raising remaining concerns. I have reviewed your responses to these remaining concerns and consider that most of the issues have been adequately addressed.

Before proceeding to a final decision, please make a minor editorial revision regarding the description of sense of coherence. In your response letter, you provided evidence that SOC is relatively stable over time, including findings from a Japanese panel study. However, the corresponding text added to the manuscript is rather brief. Please incorporate this supporting evidence more explicitly into the manuscript, while also retaining the limitation that baseline SOC may still be influenced by emotional status or major life events.

No further external review is required at this stage. Please submit a clean revised manuscript and a brief response indicating where this change has been made.

I look forward to receiving your revised manuscript.

[Note: HTML markup is below. Please do not edit.]

[NOTE: If reviewer comments were submitted as an attachment file, they will be attached to this email and accessible via the submission site. Please log into your account, locate the manuscript record, and check for the action link "View Attachments". If this link does not appear, there are no attachment files.]

To ensure your figures meet our technical requirements, please review our figure guidelines: https://journals.plos.org/plosone/s/figures

You may also use PLOS’s free figure tool, NAAS, to help you prepare publication quality figures: https://journals.plos.org/plosone/s/figures#loc-tools-for-figure-preparation.

NAAS will assess whether your figures meet our technical requirements by comparing each figure against our figure specifications.

# Author comment: Author response to Decision Letter 3

**DOI:** 10.1371/journal.pone.0355726.r006

23 Jul 2026

Dear editor,

Thank you for your helpful suggestion. We have expanded the description of the temporal stability of SOC in the Methods section (lines 117–120, page 8 of the revised manuscript) by adding evidence from a three-wave Japanese panel study, including the invariance of the factor structure of the SOC3-UTHS across time and stability coefficients of 0.69 and 0.70 between consecutive annual assessments. We have also retained the limitation in the Discussion (lines 334–337, page 25 of the revised manuscript) that baseline SOC may have been influenced by participants’ emotional status or major life events.

Sincerely,

Yosuke Yamamoto, MD, PhD

Department of Healthcare Epidemiology, Graduate School of Medicine, Kyoto University

54 Kawahara-cho, Syogoin, Sakyo-ku, Kyoto 606-8501, Japan

E-mail: yamamoto.yosuke.5n@kyoto-u.ac.jp

Attachment Submitted filename: point_by_point_response 0723.docx

# Editor report: Decision Letter 3

**DOI:** 10.1371/journal.pone.0355726.r007

**Authors:** Yujiro Kuroda

26 Jul 2026

Association between sense of coherence and loss of independence or death in older adults: Locomotive Syndrome and Health Outcomes in Aizu Cohort Study

PONE-D-25-68194R3

Dear Dr. Yamamoto,

We’re pleased to inform you that your manuscript has been judged scientifically suitable for publication and will be formally accepted for publication once it meets all outstanding technical requirements.

Within one week, you’ll receive an e-mail detailing the required amendments. When these have been addressed, you’ll receive a formal acceptance letter and your manuscript will be scheduled for publication.

An invoice will be generated when your article is formally accepted. Please note, if your institution has a publishing partnership with PLOS and your article meets the relevant criteria, all or part of your publication costs will be covered. Please make sure your user information is up-to-date by logging into Editorial Manager at Editorial Manager® and clicking the ‘Update My Information' link at the top of the page. For questions related to billing, please contact billing support.

If your institution or institutions have a press office, please notify them about your upcoming paper to help maximize its impact. If they’ll be preparing press materials, please inform our press team as soon as possible -- no later than 48 hours after receiving the formal acceptance. Your manuscript will remain under strict press embargo until 2 pm Eastern Time on the date of publication. For more information, please contact onepress@plos.org.

Kind regards,

Yujiro Kuroda

Academic Editor

PLOS One

Additional Editor Comments (optional):

Reviewers' comments:

# Editor report: Acceptance letter

**DOI:** 10.1371/journal.pone.0355726.r008

**Authors:** Yujiro Kuroda

PONE-D-25-68194R3

PLOS One

Dear Dr. Yamamoto,

I'm pleased to inform you that your manuscript has been deemed suitable for publication in PLOS One. Congratulations! Your manuscript is now being handed over to our production team.

At this stage, our production department will prepare your paper for publication. This includes ensuring the following:

* All references, tables, and figures are properly cited

* All relevant supporting information is included in the manuscript submission,

* There are no issues that prevent the paper from being properly typeset

You will receive further instructions from the production team, including instructions on how to review your proof when it is ready. Please keep in mind that we are working through a large volume of accepted articles, so please give us a few days to review your paper and let you know the next and final steps.

Lastly, if your institution or institutions have a press office, please let them know about your upcoming paper now to help maximize its impact. If they'll be preparing press materials, please inform our press team within the next 48 hours. Your manuscript will remain under strict press embargo until 2 pm Eastern Time on the date of publication. For more information, please contact onepress@plos.org.

You will receive an invoice from PLOS for your publication fee after your manuscript has reached the completed accept phase. If you receive an email requesting payment before acceptance or for any other service, this may be a phishing scheme. Learn how to identify phishing emails and protect your accounts at https://explore.plos.org/phishing.

If we can help with anything else, please email us at customercare@plos.org.

Thank you for submitting your work to PLOS One and supporting open access.

Kind regards,

PLOS ONE Editorial Office Staff

on behalf of

Dr. Yujiro Kuroda

Academic Editor

PLOS One
